# Impact of the EURO-PERISTAT Reports on obstetric management: a difference-in-regression-discontinuity analysis

**DOI:** 10.1093/eurpub/ckad013

**Published:** 2023-02-20

**Authors:** Leonie A Daalderop, Jasper V Been, Eric A P Steegers, Loes C M Bertens

**Affiliations:** Department of Obstetrics and Gynaecology, Erasmus MC, University Medical Centre Rotterdam, Rotterdam, The Netherlands; Department of Obstetrics and Gynaecology, Erasmus MC, University Medical Centre Rotterdam, Rotterdam, The Netherlands; Division of Neonatology, Department of Paediatrics, Erasmus MC—Sophia Children’s Hospital, University Medical Centre Rotterdam, Rotterdam, The Netherlands; Department of Public Health, Erasmus MC, University Medical Centre Rotterdam, Rotterdam, The Netherlands; Department of Obstetrics and Gynaecology, Erasmus MC, University Medical Centre Rotterdam, Rotterdam, The Netherlands; Department of Obstetrics and Gynaecology, Erasmus MC, University Medical Centre Rotterdam, Rotterdam, The Netherlands

## Abstract

**Background:**

Population health monitoring, such as perinatal mortality and morbidity rankings published by the European Perinatal Health (EURO-PERISTAT) reports may influence obstetric care providers’ decision-making and professional behaviour. We investigated short-term changes in the obstetric management of singleton term deliveries in the Netherlands following publication of the EURO-PERISTAT reports in 2003, 2008 and 2013.

**Methods:**

We used a quasi-experimental difference-in**-**regression-discontinuity approach. National perinatal registry data (2001–15) was used to compare obstetric management at delivery in four time windows (1, 2, 3 and 5 months) surrounding publication of each EURO-PERISTAT report.

**Results:**

The 2003 EURO-PERISTAT report was associated with higher relative risks (RRs) for an assisted vaginal delivery across all time windows [RR (95% CI): 1 month: 1.23 (1.05–1.45), 2 months: 1.15 (1.02–1.30), 3 months: 1.21 (1.09–1.33) and 5 months: 1.21 (1.11–1.31)]. The 2008 report was associated with lower RRs for an assisted vaginal delivery at the 3- and 5-month time windows [0.86 (0.77–0.96) and 0.88 (0.81–0.96)]. Publication of the 2013 report was associated with higher RRs for a planned caesarean section across all time windows [1 month: 1.23 (1.00–1.52), 2 months: 1.26 (1.09–1.45), 3 months: 1.26 (1.12–1.42) and 5 months: 1.19(1.09–1.31)] and lower RRs for an assisted vaginal delivery at the 2-, 3- and 5-month time windows [0.85 (0.73–0.98), 0.83 (0.74–0.94) and 0.88 (0.80–0.97)].

**Conclusions:**

This study showed that quasi-experimental study designs, such as the difference-in-regression-discontinuity approach, are useful to unravel the impact of population health monitoring on decision-making and professional behaviour of healthcare providers. A better understanding of the contribution of health monitoring to the behaviour of healthcare providers can help guide improvements within the (perinatal) healthcare chain.

## Introduction

The Netherlands has a unique perinatal healthcare system, which is based on the principle that pregnancy, childbirth, and the postpartum period are fundamentally physiological processes.^1^ At the primary care level, low-risk pregnant women are guided and supported by community midwives. If complications occur, or threaten to occur, pregnant women are referred to hospital-based obstetric care provided by obstetricians (secondary and tertiary level of care).^2^^,^^3^ Low-risk pregnant women can opt for an out-of-hospital delivery either at home, in a primary care birth centre, or in an outpatient clinic. Out-of-hospital deliveries are common in the Netherlands: 19.1% of all nulliparous women and 42.7% of all multiparous women had an out-of-hospital delivery in 2019, compared to less than 1% in most other European countries.^4^^,^^5^ In the UK, where an out-of-hospital delivery is offered as an option to low-risk pregnant women, this percentage ranged from 1.4% in Scotland to 3.7% in Wales.^5^

The publication of the European Perinatal Health (EURO-PERISTAT) reports in 2003 and 2008 sparked significant discussion regarding the safety of, and outcomes related to, the Dutch perinatal healthcare system.^6^^,^^7^ The EURO-PERISTAT reports assemble statistical information from 31 European countries on population characteristics, health and healthcare use of pregnant women and their babies. The publications in 2003 and 2008 showed that perinatal mortality and morbidity rates in the Netherlands were among the highest in Europe and had a slow temporal decline compared to other European countries.^6–8^ These findings received significant media attention, which triggered a public and political debate stimulating a policy process directed at improving perinatal health.^9^ A national Steering Group was installed in 2008, to identify and monitor potential causes of the poor perinatal health outcomes.^9^ Subsequently, a national perinatal audit system and several intervention programmes were developed to assess and address the likely underlying causes of the higher perinatal mortality and morbidity rates in the Netherlands.^10–13^ The 2013 and 2018 EURO-PERISTAT reports showed that perinatal health outcomes had improved considerably since 2008;^5^^,^^14^ the Netherlands had acquired a middle position in the European rankings for perinatal health outcomes.

Although the primary goal of the EURO-PERISTAT reports is to inform public health policies, the wide-spread media attention and social debate following publication also made obstetric care providers more aware of the need to address adverse perinatal health outcomes.^9^ In addition, several studies in the field of perinatology show that published study results have the potential to induce short-term practice changes among healthcare providers.^15^^,^^16^ As a result, we expected that publication of the EURO-PERISTAT reports influenced individual obstetric care providers to change their decision-making and professional behaviour regarding obstetric management at delivery directly following publication. However, this has not been previously evaluated in the Netherlands. Therefore, the aim of this study was to investigate whether any changes in the obstetric management of singleton term deliveries occurred in the Netherlands directly following publication of the EURO-PERISTAT reports in 2003, 2008 and 2013. We hypothesised a change towards more active obstetric management at delivery (i.e. more inductions of labour, vaginal assisted deliveries and caesarean sections) after publication of the EURO-PERISTAT reports, with the most pronounced changes expected after publication of the first report. This is because such changes have the potential to directly influence maternal and perinatal health outcomes at birth. Our hypothesis was assessed in a national quasi-experimental study using perinatal registry data and application of a difference-in-regression-discontinuity design.

## Methods

### Design and study population

We used national perinatal registry data on all singleton deliveries between 37 + 0 and 40 + 6 weeks of gestation between January 2001 and December 2015 in the Netherlands to evaluate the impact of the 2003, 2008 and 2013 EURO-PERISTAT reports on any short-term changes in obstetric management at delivery. For this purpose, three cohorts were created around the different EURO-PERISTAT reports (2001–05, 2006–10 and 2011–15). The impact of the 2018 EURO-PERISTAT report was not investigated since national perinatal registry data were not yet available for the years 2019 and 2020. Preterm and post-term deliveries were excluded, because they are more strictly guided by protocols than deliveries in the selected term period.^17^ Protocols regarding the preterm period aim to maintain pregnancy if the condition of the mother and child allows it, as the survival rates increase significantly per week of pregnancy. These protocols therefore relate to the inhibition of preterm labour and preparing the foetus to enhance survival rates.^18^ Active obstetric management during the preterm period is therefore mainly driven by expected adverse outcomes of the mother and/or her child, and fairly protocolled. Although post-term deliveries are considered to be deliveries after 42 weeks of gestation, we excluded all deliveries from 41 + 0 weeks onwards. We excluded these deliveries because pregnant women in the Netherlands can opt for an induction of labour from 41 + 0 weeks onwards,^19^ so obstetric management during this period is also guided by the personal preferences of the pregnant woman. Consequently, we expect that pregnancies in the term period are most subject to preferences of obstetric care providers, which is the reason why we selected pregnancies in the term period.

Perinatal registry data were obtained from Perined. The Perined registry contains information on more than 97% of all pregnancies in the Netherlands (Perined, www.perined.nl). Midwives, obstetricians and paediatricians/neonatologists routinely report pregnancy, delivery and neonatal data to Perined using standard report forms. Perined uses probabilistic record linkage to combine information of the different population-based Dutch Perinatal Registries (detailed information regarding the linkage procedure can be found in Méray et al.^20^)

### Outcomes

Several active obstetric management options at the start and end of a delivery were considered as outcomes. A spontaneous start and/or end of delivery was categorised as ‘no active obstetric management’. Active obstetric management options at the start of a delivery were defined as: (i) induction of labour or (ii) planned caesarean section. Active obstetric management options at the end of a delivery were defined as: (i) assisted vaginal delivery (i.e. vacuum extraction or forceps delivery) or (ii) emergency caesarean section.

### Covariates

Maternal age, parity, ethnicity and neighbourhood socioeconomic status (SES) were included in the analyses to account for heterogeneity in obstetric management at delivery between subgroups. Maternal age was categorised into five age groups: <20, 21–25, 26–30 (reference category), 31–35 and ≥36 years. Parity was dichotomised into nulliparous and multiparous women (reference category). Ethnicity was dichotomised into European (reference category) and non-European as the quality of the registration in the Perined database did not allow further differentiation. Lastly, neighbourhood SES was categorised into three groups: below the 20th percentile (most deprived neighbourhoods), between the 20th and 80th percentile, and above the 80th percentile (most affluent neighbourhoods, reference category). The neighbourhood SES score was based on an area-level SES indicator at the four-digit postal code level constructed by the Netherlands Institute for Social Research (SCP, www.scp.nl). This indicator is composed based on a principal component analysis of (i) mean annual income per household, (ii) percentage of households with a low income, (iii) percentage of households with a low education and (iv) percentage of unemployed inhabitants.^21^

### Statistical analysis

First, maternal, neonatal and delivery characteristics were compared between the three cohorts surrounding the EURO-PERISTAT reports to identify major differences over time. Subsequently, prevalence rates of the different obstetric management options at the start and end of a delivery were plotted across the period spanning one year before to one year after the publication of each of the EURO-PERISTAT reports (published 27 November 2003, 15 December 2008 and 27 May 2013) to observe changes over years.

**Figure 1:**
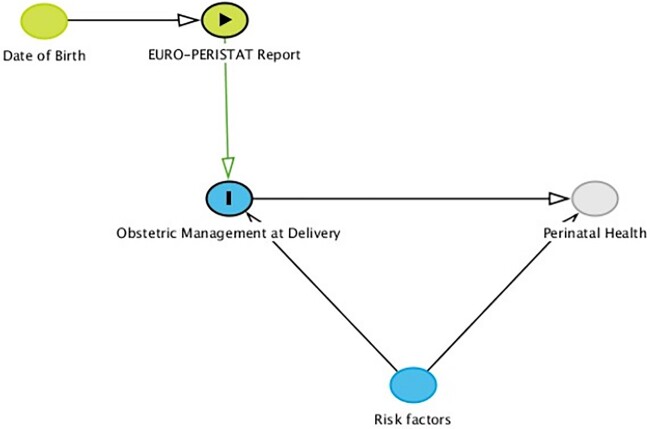
Directed acyclic graph. A directed acyclic graph (DAG) can be used to map a priori assumptions surrounding a causal question of interest. The idea of the regression discontinuity (RD) design is to compare deliveries close to the threshold (EURO-PERISTAT publication). Those with a date of birth just above the threshold should be comparable to the ones with a date of birth just below the threshold. Important is that none of the other variables in the model should exhibit any discontinuity around the publication of the EURO-PERISTAT reports. In addition, only locally valid effects of the EURO-PERISTAT reports are obtained, because deliveries further away from the threshold (i.e. time of publication), will cease to be really comparable. So, in this study date of birth determines whether a delivery is exposed or not to the information published in the EURO-PERISTAT reports. Information in the EURO-PERISTAT reports may result in alterations in obstetric management at delivery, which may affect perinatal health outcomes. To provide a valid RD analysis it is important that additional explanatory variables (e.g. risk factor in the DAG) do not suddenly alter around the threshold, because we will compare deliveries slightly left and right of the threshold. We therefore need to make sure that these variables would confound not our estimate. To do so, we accounted for variations in maternal characteristics that may cause heterogeneity in the estimated impact of the EURO-PERISTAT reports, by adjusting for maternal age, parity, ethnicity and neighbourhood SES

We used a difference-in-regression-discontinuity analysis,^22^ fitting a generalised linear Quasi-Poisson regression model, to study the short-term impact of the three EURO-PERISTAT reports on obstetric management at delivery. This quasi-experimental technique can be used when the exposure of interest is assigned by the value of a continuously measured random variable and whether that variable lies above (or below) a cut-off value. Provided that subjects cannot precisely manipulate the value of this variable, assignment of the exposure is nearly as random for observations close to the cut-off, and valid causal effects can be identified. In this study, date of birth is the assignment variable that determines whether a delivery is ‘exposed’ or ‘not exposed’ to the information published in the EURO-PERISTAT reports (i.e. publication date of the reports is the cut-off variable) (figure 1). Deliveries immediately above and below the cut-off will be similar, in expectation, on all observed and unobserved characteristics, just as in a randomised controlled trial. Causal effects can be estimated by comparing outcomes in these deliveries. To enable a more strict distinction between exposed and unexposed deliveries, the 2 weeks following the publication of the EURO-PERISTAT reports were considered a transition period and therefore censored in the analyses. All three reports were announced via a press release in the week of the publication and received substantial media attention. As such, we assume that the vast majority of obstetric care providers were aware of the key findings presented in the EURO-PERISTAT publications within two weeks after the publication.

We evaluated changes in obstetric management at delivery in four time windows in separate analyses: 1, 2, 3 and 5 months before and after the publication dates. With the use of these relatively short-time windows, we exclude other interventions or major influences that might affect obstetric management at delivery, allowing us to assume that any observed change was due to the specific EURO-PERISTAT publication.

The analyses accounted for underlying temporal trends, time-variant factors and seasonality that might affect obstetric management at delivery by comparing obstetric management in the time-windows surrounding publication of the EURO-PERISTAT reports in 2003, 2008 and 2013 to the exact same time windows in the 2 years before and 2 years after the publication dates. These previous and subsequent cohorts in the model act as control periods to account for any existing temporal trends that occur every year around the cut-off date. By doing so, the change in outcome estimated at the cut-off in the year of interest will be adjusted to the observed changes at the same time point, but in a different year. This is facilitated by the difference-in-regression-discontinuity extension of the regression discontinuity design. In addition, we accounted for variations in maternal characteristics that may cause heterogeneity in the estimated impact of the EURO-PERISTAT reports, by adjusting for maternal age, parity, ethnicity and neighbourhood SES.

All the conditions for a valid regression discontinuity analysis were met:^23^ (i) the cut-off value was known (publication dates of the different EURO-PERISTAT reports), (ii) the assignment variable (date of birth, measured in days) is continuous near the cut-off and not affected by publication of the EURO-PERISTAT reports (Supplementary figures S1–S3), (iii) outcomes are observed for all pregnancies (independent of the exposure), (iv) covariates are not discontinuous around the threshold, demonstrating comparability (Supplementary figures S4–S6) and (v) there is visual confirmation of the discontinuity, suggesting an effect of the EURO-PERISTAT publications on obstetric management at delivery (Supplementary figures S7–S24).

Lastly, sensitivity analyses were performed to investigate whether the impact of the EURO-PERISTAT publications was potentially too immediate to have been captured by our primary analytical approach. To check this, all analyses were also performed without omitting the 2 weeks directly following the publication dates.

### Ethical approval

The data used in this study are national registry data and no formal ethical approval was needed for these analyses according to Dutch law. Approval for the use of the data was obtained from the board of directors from Perined (protocol 16.37).

## Results

**Figure 2:**
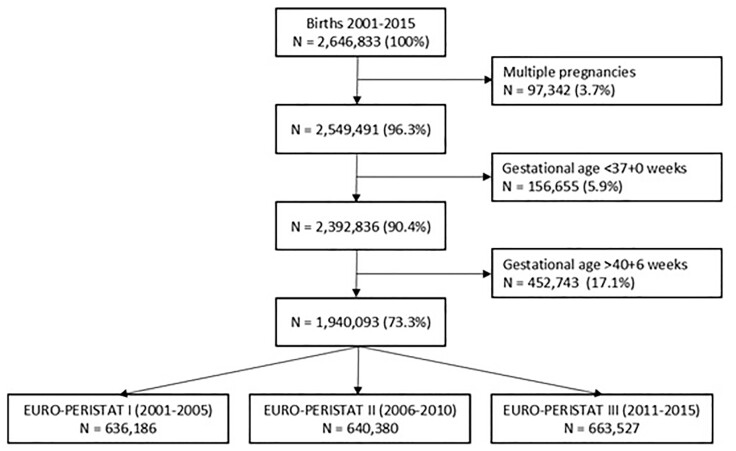
Flowchart of study sample

Between January 2001 and December 2015, 2 646 833 births were registered with Perined. Of these, 97 342 multiple pregnancies were excluded. Next, records with a gestational age <37 + 0 weeks (*n* = 156 655) and >40 + 6 weeks (*n* = 452 743) were excluded. There were no missing data regarding obstetric management at delivery, yielding a sample size of 1 940 093 singleton births for this study, divided in three cohorts around the publication dates of the different EURO-PERISTAT reports (figure 2).

In all three cohorts, the majority of the deliveries started and ended spontaneously (table 1), but the proportion that started spontaneously decreased over time. The incidence of labour inductions increased sharply between 2001 and 2015, from 11.6 to 19.5%. Incidences regarding management options at the end of a delivery remained relatively stable over time (table 1 and Supplementary figures S25 and S26).

**Table 1:** Baseline characteristics of the three cohorts created around the EURO-PERISTAT 2003 (27th November), 2008 (15th December) and 2013 (27th May) reports

	Cohort 1 (2001–05)	Cohort 2 (2006–10)	Cohort 3 (2011–15)
	*n* = 636 186	*n* = 640 380	*n* = 663 527
Maternal characteristics, *n* (%)			
Maternal age (years)			
≤20	17 495 (2.8)	15 378 (2.4)	13 534 (2.0)
21–25	80 130 (12.6)	86 282 (13.5)	88 128 (13.3)
26–30	207 194 (32.6)	213 833 (33.4)	231 465 (34.9)
31–35	239 860 (37.8)	220 849 (34.5)	229 436 (34.6)
≥36	90 479 (14.2)	103 561 (16.2)	100 760 (15.2)
Parity			
Nulliparous	317 714 (49.9)	323 305 (50.5)	338 352 (51.0)
Multiparous	318 429 (50.1)	317 049 (49.5)	325 146 (49.0)
Ethnicity			
European	525 378 (83.5)	511 062 (80.3)	513 614 (77.9)
Non-European	103 441 (16.5)	125 328 (19.7)	145 326 (22.1)
Neighbourhood socioeconomic status			
<p20	120 024 (19.0)	124 925 (19.6)	136 295 (20.6)
p20–p80	373 580 (59.1)	369 888 (58.0)	384 204 (58.1)
>p80	138 927 (22.0)	142 709 (22.4)	140 469 (21.3)
Delivery characteristics, *n* (%)			
Start of delivery			
Spontaneous	518 354 (81.5)	506 023 (79.0)	482 476 (72.7)
Induction	73 491 (11.6)	90 157 (14.1)	129 162 (19.5)
Planned caesarean section^a^	44 341 (7.0)	44 200 (6.9)	51 889 (7.8)
End of delivery			
Spontaneous vaginal delivery	488 210 (76.7)	491 046 (76.7)	508 142 (76.6)
Assisted vaginal delivery	62 311 (9.8)	59 165 (9.2)	54 315 (8.2)
Emergency caesarean section	41 324 (6.5)	45 969 (7.2)	49 180 (7.4)
Planned caesarean section^a^	44 341 (7.0)	44 200 (6.9)	51 889 (7.8)
Neonatal characteristics, *n* (%)			
Perinatal mortality^b^	2075 (0.3)	1576 (0.2)	1140 (0.2)
Small for gestational age	74 901 (11.8)	68 892 (10.8)	69 531 (10.5)

a Planned caesarean section is part of both the start and end of a delivery.

b Perinatal mortality is defined as death occurring between 22 weeks gestational age and 7 days after birth.

### Changes in obstetric management at the start of a delivery

Publication of both the 2003 and 2008 EURO-PERISTAT reports were not associated with clear changes in obstetric management at the start of a delivery (table 2). After publication of the 2013 EURO-PERISTAT report, a more pro-active management was observed, with higher relative risks (RRs) for a planned caesarean section across all time windows surrounding publication [RRs: 1 month 1.23 (95% CI 1.00–1.52), 2 months 1.26 (95% CI 1.09–1.45), 3 months 1.26 (95% CI 1.12–1.42) and 5 months 1.19 (95% CI 1.09–1.31)].

**Table 2:** Impact of the EURO-PERISTAT 2003 (27th November), 2008 (15th December) and 2013 (27th May) reports on obstetric management at the start and end of a delivery across different time windows before and after publication

	Time window			
	1 month	2 months	3 months	5 months
	RR (95% CI)^a^	RR (95% CI)^a^	RR (95% CI)^a^	RR (95% CI)^a^
Cohort 1: EURO-PERISTAT 2003 analysis^b^	*n* = 126 498	*n* = 232 762	*n* = 341 005	*n* = 550 155
Start of delivery				
Spontaneous	0.98 (0.96–1.01)	0.99 (0.97–1.01)	0.99 (0.98–1.01)	1.00 (0.99–1.01)
Induction of labour	1.07 (0.92–1.23)	1.05 (0.94–1.18)	1.05 (0.98–1.17)	1.03 (0.96–1.11)
Planned caesarean section	1.10 (0.91–1.33)	0.99 (0.86–1.14)	0.96 (0.86–1.09)	0.97 (0.88–1.06)
End of delivery				
Spontaneous vaginal delivery	0.98 (0.95–1.01)	0.99 (0.97–1.02)	0.99 (0.97–1.01)	0.98 (0.97–1.00)
Assisted vaginal delivery	**1.23 (1.05**–**1.45)**	**1.15 (1.02**–**1.30)**	**1.21 (1.09**–**1.33)**	**1.21 (1.11**–**1.31)**
Emergency caesarean section	0.83 (0.68–1.01)	0.89 (0.76–1.03)	**0.87 (0.77**–**0.98)**	0.98 (0.89–1.08)
Cohort 2: EURO-PERISTAT 2008 analysis^b^	*n* = 103 094	*n* = 210 344	*n* = 313 254	*n* = 528 828
Start of delivery				
Spontaneous	1.03 (1.00–1.06)	1.01 (0.98–1.03)	1.00 (0.98–1.02)	1.00 (0.99–1.02)
Induction of labour	0.86 (0.74–1.00)	0.93 (0.84–1.03)	1.05 (0.88–1.05)	0.98 (0.92–1.05)
Planned caesarean section	0.95 (0.76–1.20)	1.08 (0.92–1.26)	1.05 (0.92–1.19)	1.01 (0.92–1.12)
End of delivery				
Spontaneous vaginal delivery	1.03 (0.99–1.06)	1.01 (0.99–1.03)	1.02 (1.00–1.04)	**1.02 (1.00**–**1.03)**
Assisted vaginal delivery	0.85 (0.70–1.03)	0.89 (0.78–1.02)	**0.86 (0.77**–**0.96)**	**0.88 (0.81**–**0.96)**
Emergency caesarean section	0.98 (0.79–1.22)	0.98 (0.85–1.15)	0.96 (0.84–1.08)	0.95 (0.86–1.04)
Cohort 3: EURO-PERISTAT 2013 analysis^b^	*n* = 117 982	*n* = 229 158	*n* = 335 513	*n* = 551 631
Start of delivery				
Spontaneous	0.98 (0.94–1.02)	**0.97 (0.94**–**0.99)**	**0.97 (0.95**–**0.99)**	**0.98 (0.96**–**0.99)**
Induction of labour	0.98 (0.86–1.11)	1.04 (0.95–1.14)	1.04 (0.96–1.11)	1.02 (0.96–1.08)
Planned caesarean section	**1.23 (1.00**–**1.52)**	**1.26 (1.09**–**1.45)**	**1.26 (1.12**–**1.42)**	**1.19 (1.09**–**1.31)**
End of delivery				
Spontaneous vaginal delivery	0.98 (0.94–1.01)	0.99 (0.97–1.01)	0.98 (0.97–1.00)	0.99 (0.97–1.00)
Assisted vaginal delivery	1.05 (0.84–1.30)	**0.85 (0.73**–**0.98)**	**0.83 (0.74**–**0.94)**	**0.88 (0.80**–**0.97)**
Emergency caesarean section	0.95 (0.77–1.18)	1.01 (0.87–1.17)	1.09 (0.97–1.23)	1.09 (0.99–1.20)

^a^ All analyses were adjusted for maternal age, parity, ethnicity, and neighbourhood socioeconomic status and significant results are highlighted in bold.

b The reference group is the obstetric management option in the same time window before publication (e.g. RR for induction of labour in the 1-month time window shows the risks for a labour induction 1 month after the publication compared to 1 month before the publication).

### Changes in obstetric management at the end of a delivery

Publication of the 2003 EURO-PERISTAT report was associated with a more pro-active management at the end of a delivery; higher RRs for an assisted vaginal delivery were observed across all time windows [RRs: 1 month 1.23 (95% CI 1.05–1.45), 2 months 1.15 (95% CI 1.02–1.30), 3 months 1.21 (95% CI 1.09–1.33) and 5 months 1.21 (95% CI 1.11–1.31)]. Conversely, the 2008 report was associated with lower RRs for an assisted vaginal delivery in the 3- and 5-month time windows [RRs: 3 months 0.86 (95% CI 0.77–0.96) and 5 months 0.88 (95% CI 0.81–0.96)]. Publication of the 2013 EURO-PERISTAT publication was also associated with lower RRs for an assisted vaginal delivery at each but the 1-month time window [RRs: 1 month 1.05 (95% CI 0.84–1.30), 2 months 0.85 (95% CI 0.73–0.98), 3 months 0.83 (95% CI 0.74–0.94) and 5 months 0.88 (95% CI 0.80–0.97)].

### Sensitivity analyses

The sensitivity analyses, without omitting 2 weeks after the publication date of the EURO-PERISTAT reports, produced similar associations, both in terms of direction and magnitude of the associations (Supplementary table S1).

## Discussion

### Key results

With the use of a difference-in-regression-discontinuity design, we observed direct changes in obstetric management of singleton term deliveries in the Netherlands following publication of the EURO-PERISTAT reports in 2003, 2008 and 2013. The observed changes were indicative for a more pro-active management after publication of the 2003 and 2013 report, but not after the 2008 report.

### Strengths and limitations

The main strengths of this study are the use of a very large and nationally representative database, and the quasi-experimental study design. The difference-in-regression-discontinuity approach takes temporal variability, unmeasured confounding and time-variant factors into account, which reduces bias. An additional advantage of the difference-in-regression-discontinuity design over a regression-discontinuity approach is that this design accounts for yearly seasonal trends. We accounted for such trends by augmenting our sample and including deliveries around the cut-off in the 2 years before and after the publication dates.

Despite these strengths, several limitations merit discussion. First, although changes in obstetric management at delivery were observed, there was insufficient power to assess the impact of these changes on perinatal mortality within the limited time windows, as perinatal mortality is a rare outcome. Second, the difference-in-regression-discontinuity design limits the interpretation of the findings to a short-term period only.^24^^,^^25^ The unexpected findings presented in the first EURO-PERISTAT report triggered a strong response that persisted for at least 5 months. Although it is expected that these direct observed changes in obstetric management will fade over time, a process directed at policy changes throughout the entire perinatal healthcare chain has been initiated as a result of the EURO-PERISTAT publications, which has led to long-term changes in practice.^9^ These policy changes initiated following the first publication may also explain the absence of an additional move towards more pro-active delivery management after the second EURO-PERISTAT report. Third, we were able to take only a selection of maternal characteristics that may have affected obstetric management at delivery into account. For example, we lacked information on known risk factors for adverse perinatal health outcomes that may also have an impact on obstetric management at delivery, such as body mass index (BMI), gestational diabetes and pre-eclampsia.^26^^,^^27^ However, it is unlikely that the these risk factors were unevenly distributed around the publication dates. Lastly, we were unable to determine the proportion of obstetric care providers that were aware of the different EURO-PERISTAT publications. Since all three EURO-PERISTAT publications were announced via a press release by, among others, the national professional colleges of midwives and gynaecologists and received substantial (inter)national media attention, we assume that the vast majority of obstetric care providers were aware of the key findings presented in the EURO-PERISTAT publications.

### Interpretation

We are unaware of other studies that investigated the impact of the EURO-PERISTAT publications on daily obstetric practice. This is remarkable, as the main aim of the EURO-PERISTAT reports is to provide valid and reliable indicators that can be used by countries to monitor, evaluate and compare their perinatal health outcomes to other European countries.^28^ Such comprehensive population health monitoring is a well-known method to inform public health policies.^29^ In addition, our study suggests that population health monitoring can also influence healthcare providers’ decision-making and professional behaviour. We observed changes in obstetric management both at the start and end of a delivery occurring shortly after publication of the different reports. Obstetric management at delivery was more pro-active after publication of the 2003 and 2013 report, but not after the 2008 report. It is unclear to us why the observed changes differed per EURO-PERISTAT report. Increased awareness of the importance of adverse perinatal health outcomes caused by the wide-spread media attention and intense social debate following publication of the EURO-PERISTAT reports is the most likely underlying mechanism for the observed changes after publication of the 2003 and 2013 report. To enable a more strict distinction between exposed and unexposed deliveries, the 2 weeks following the publication of the EURO-PERISTAT reports were considered a transition period and therefore censored in the analyses. However, the main and sensitivity analyses produced similar associations, both in terms of direction and magnitude of the associations, suggesting that there was no transition period.

In the field of population health monitoring, the common assumption is that there are two communities: the evidence producers (e.g. health information analysts, university researchers, national and regional public health institutes) and the evidence users (e.g. policy-makers).^29^ Next to our findings, other studies in the field of perinatology also show that published study results can induce immediate and sustained behavioural change among healthcare providers after publication and even before the results are translated into guidelines.^15^^,^^16^ To better understand how published study results induce such behavioural changes, we call for more research into the impact of health monitoring research on the attitude and actions of healthcare providers. With the use of quasi-experimental study designs, such as the difference-in-regression-discontinuity design used in this study, the impact of population health monitoring on the decision-making and professional behaviour of the individual healthcare provider can effectively be evaluated. Next to quasi-experimental study designs, future research should also focus on the underlying mechanisms for the observed professional behavioural changes. Qualitative research methods such as interviews and focus groups can help to understand which information prompt healthcare providers to alter their behaviour.

To conclude, population health monitoring reports, like those produced by EURO-PERISTAT, not only have an impact on public health policies but also on the individual healthcare provider. This impact is observed in direct professional behavioural changes following publication of the EURO-PERISTAT reports. Quasi-experimental study designs, such as the difference-in-regression-discontinuity approach, are useful to unravel the impact of population health monitoring on decision-making and professional behaviour of healthcare providers. A better understanding of the contribution of health monitoring to the behaviour of healthcare providers can help accelerate future improvements within the healthcare chain both nationally and internationally.

## Supplementary Material

ckad013_Supplementary_Data

## Data Availability

Data is owned by a third party. The data underlying this article were provided by Perined under licence / by permission. Data will be shared on request to the corresponding author with permission of Perined.
