# Supplementary material for: Impact of the EURO-PERISTAT Reports on obstetric management: a difference-in-regression-discontinuity analysis
Source: Eur J Public Health. 2023 Feb 20;33(2):342–8. doi: 10.1093/eurpub/ckad013 (PMC10066490; doi:10.1093/eurpub/ckad013)
Supplement: ckad013_Supplementary_Data [file ckad013_supplementary_data.docx]

**Supplementary Material**

**Supplementary figures 1-3.** Histograms of the assignment variable, showing that date of birth measured in days is continuous near the publication dates of the different EURO-PERISTAT reports (November 27^th^, 2003, December 15^th^, 2008, and May 27^th^, 2013) and is not affected by the publication of the reports.

*
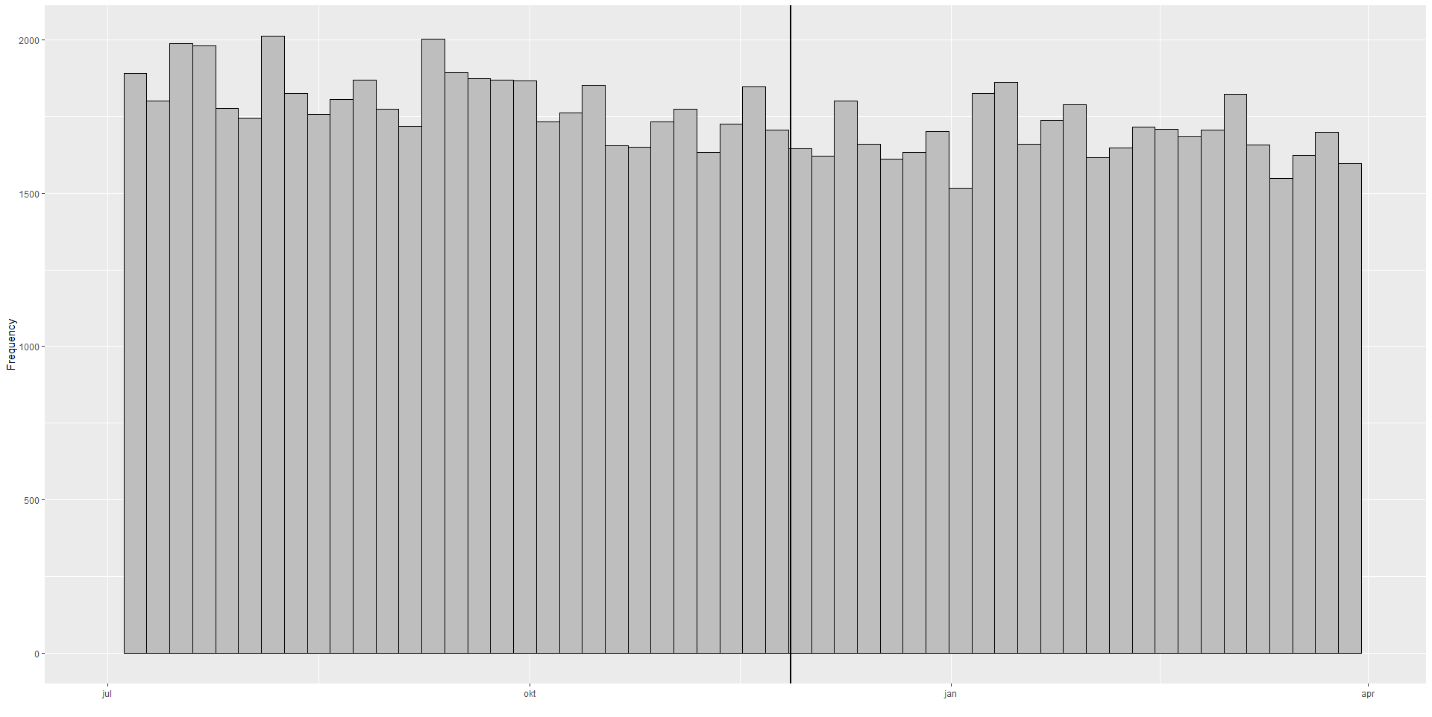
****Supplementary figure 1.*** Histogram of the 2003 EURO-PERISTAT report, showing the amount of births per day before and after the publication date (November 27^th^, 2003).


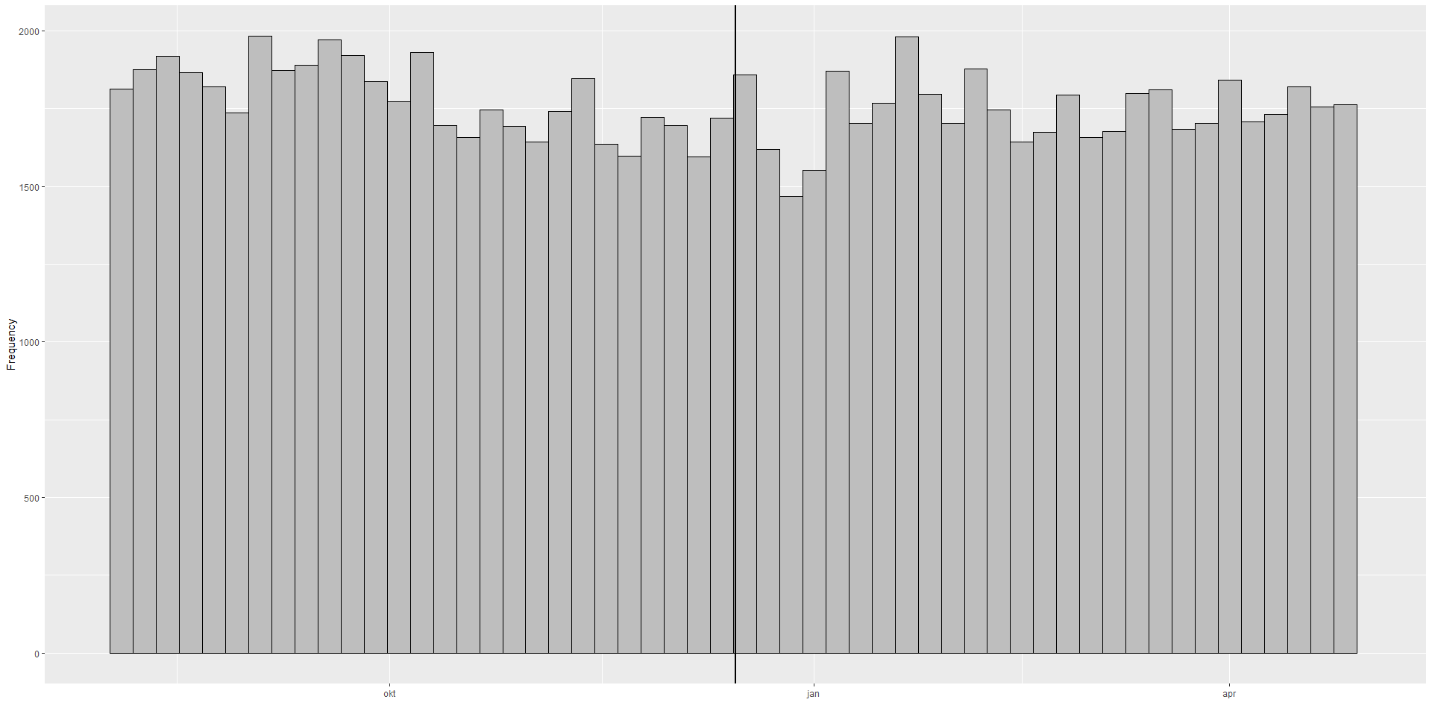
***Supplementary figure 2.*** Histogram of the 2008 EURO-PERISTAT report, showing the amount of births per day before and after the publication date (December 15^th^, 2008).

***Supplementary figure 3.*** Histogram of the 2013 EURO-PERISTAT report, showing the amount of births per day before
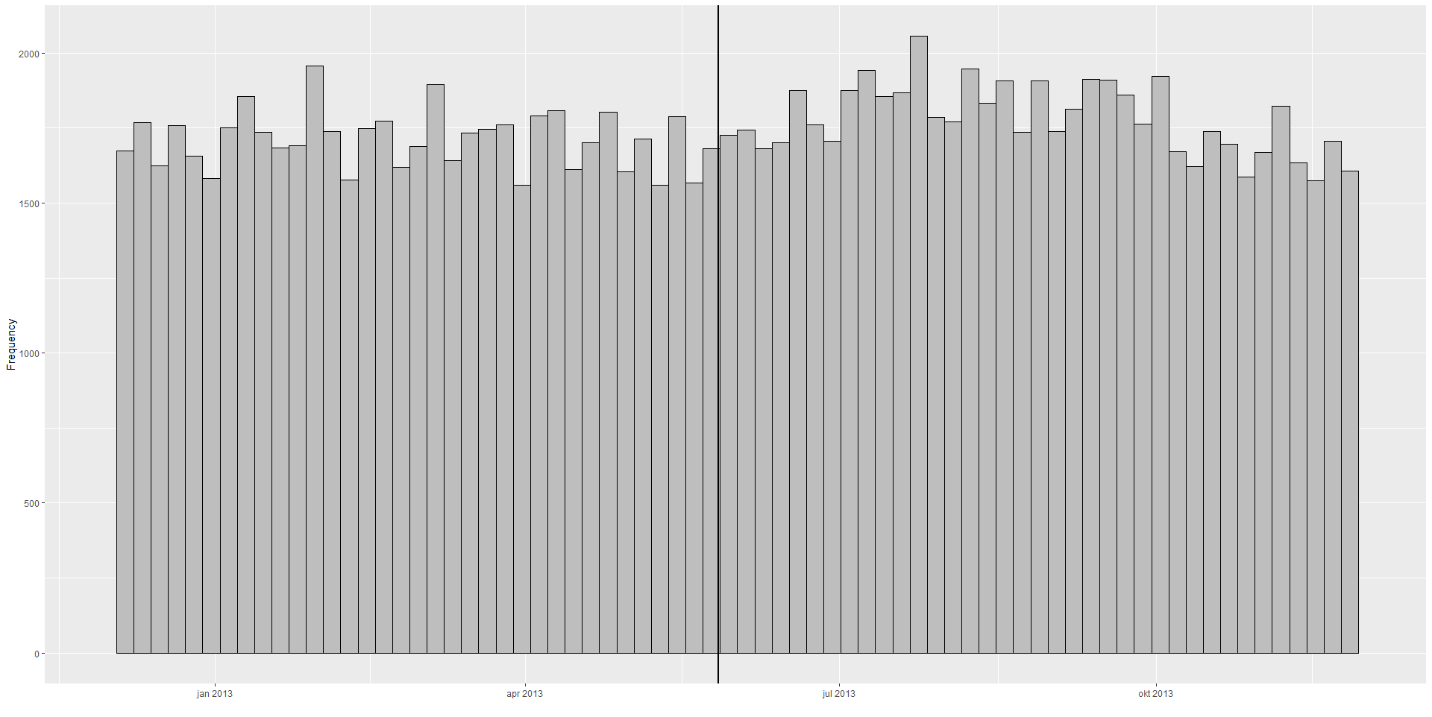
and after the publication date (May 27^th^, 2013).

**Supplementary figures 4-6.** Daily mean maternal age plotted against the assignment variable (date of birth). Maternal age is used to demonstrate comparability of the covariates in the exposed and unexposed group around the cut-off. Figures of the other covariates (parity, ethnicity, and neighbourhood socioeconomic status) are available upon request.


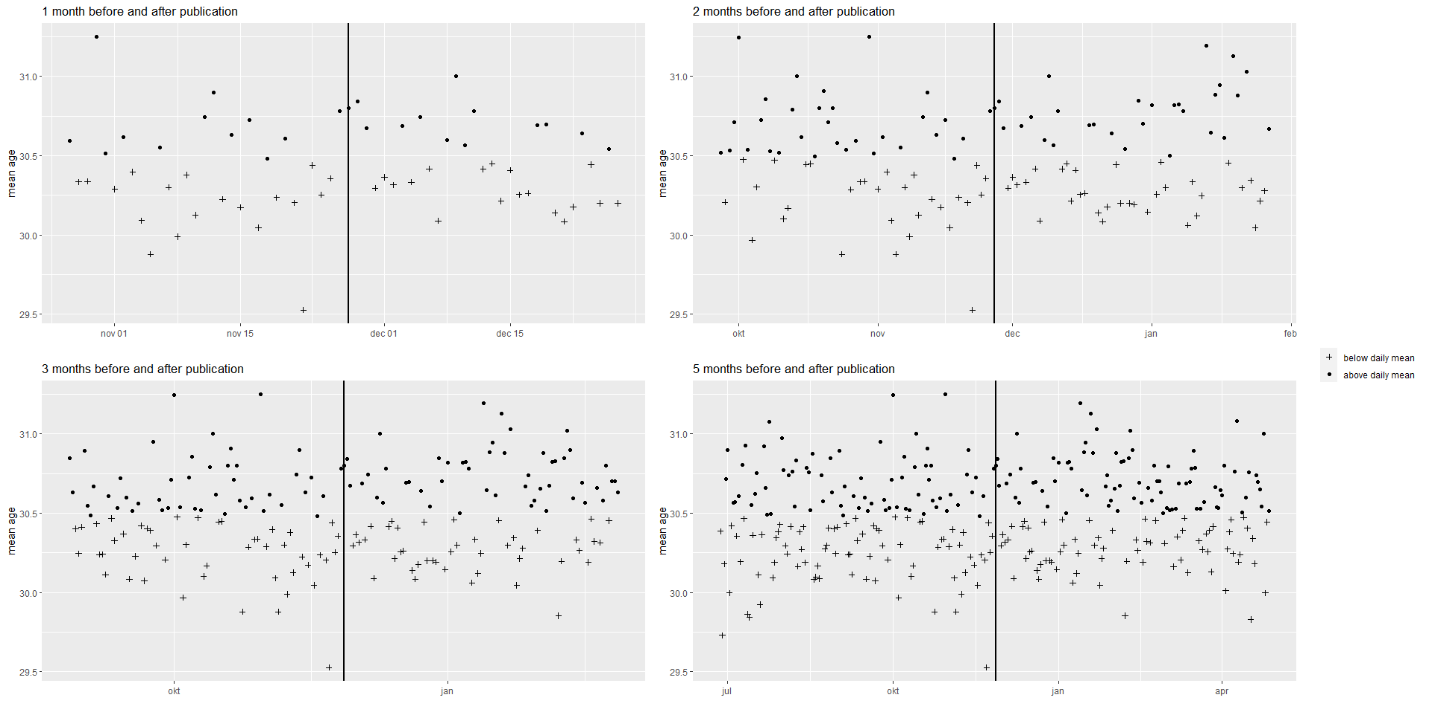
***Supplementary figure 4.*** 2003 EURO-PERISTAT report.

***Supplementary figure 5.*** 2008 EURO-PERISTAT report.


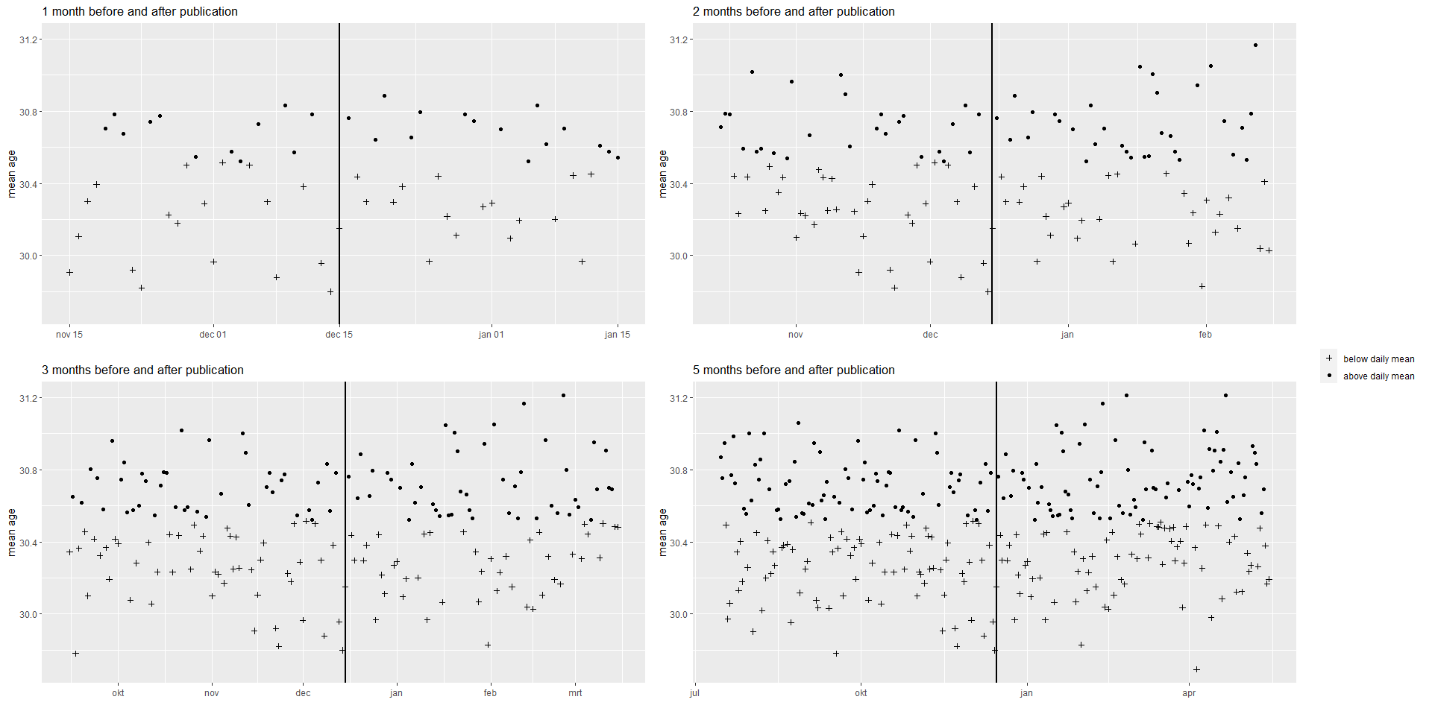


***Supplementary figure 6.*** 2013 EURO-PERISTAT report.


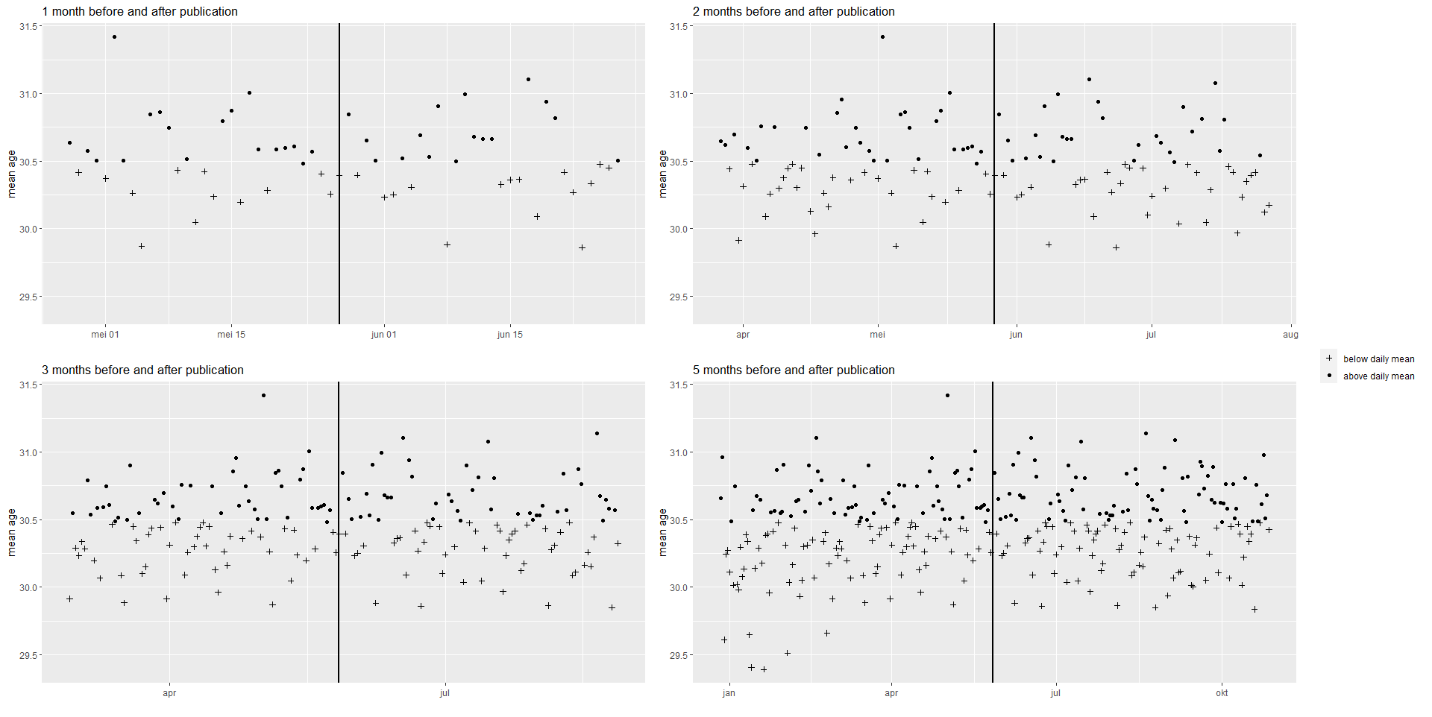


**Supplementary figures 7-24.** Visual confirmation of discontinuity.

***
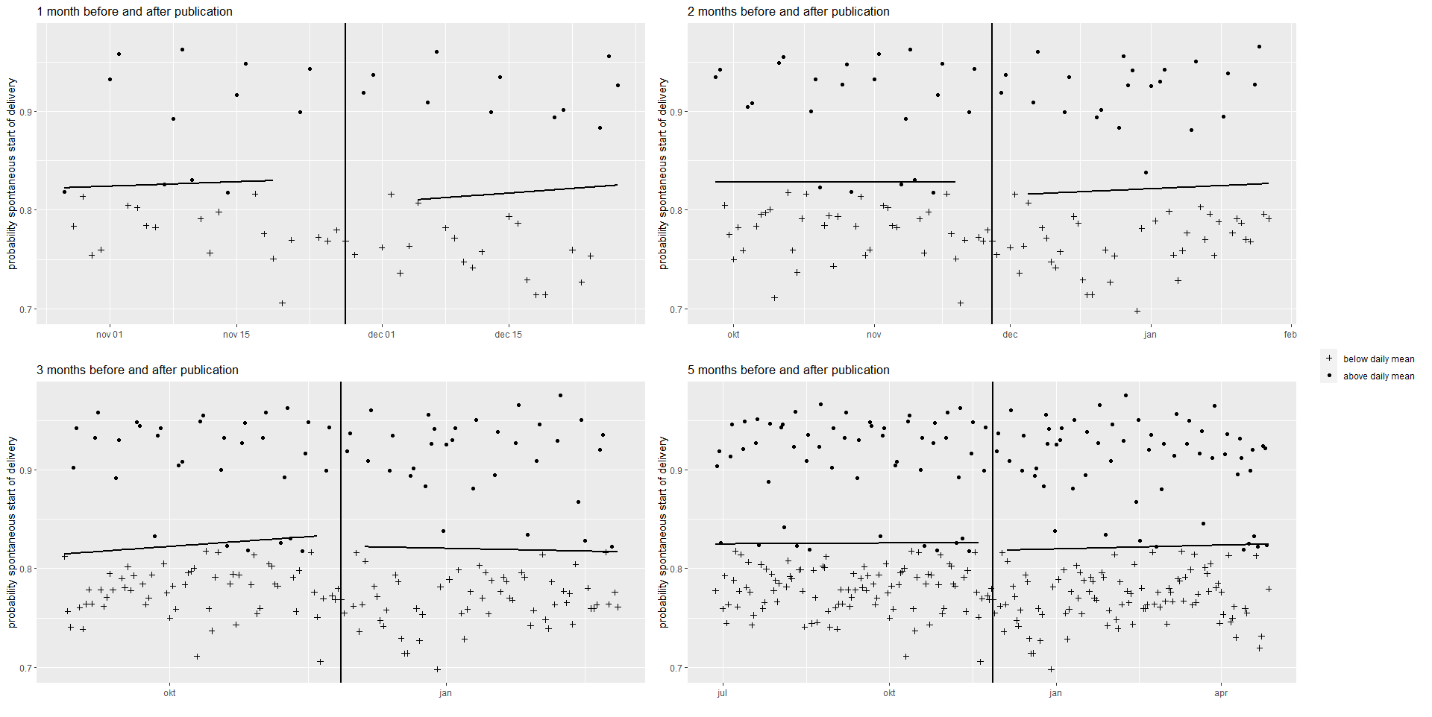
Supplementary figure 7.*** Probability of a spontaneous start of a delivery plotted against the publication date of the 2003 EURO-PERISTAT report (November 27^th^, 2003) to demonstrate discontinuity at the cut-off value.

*
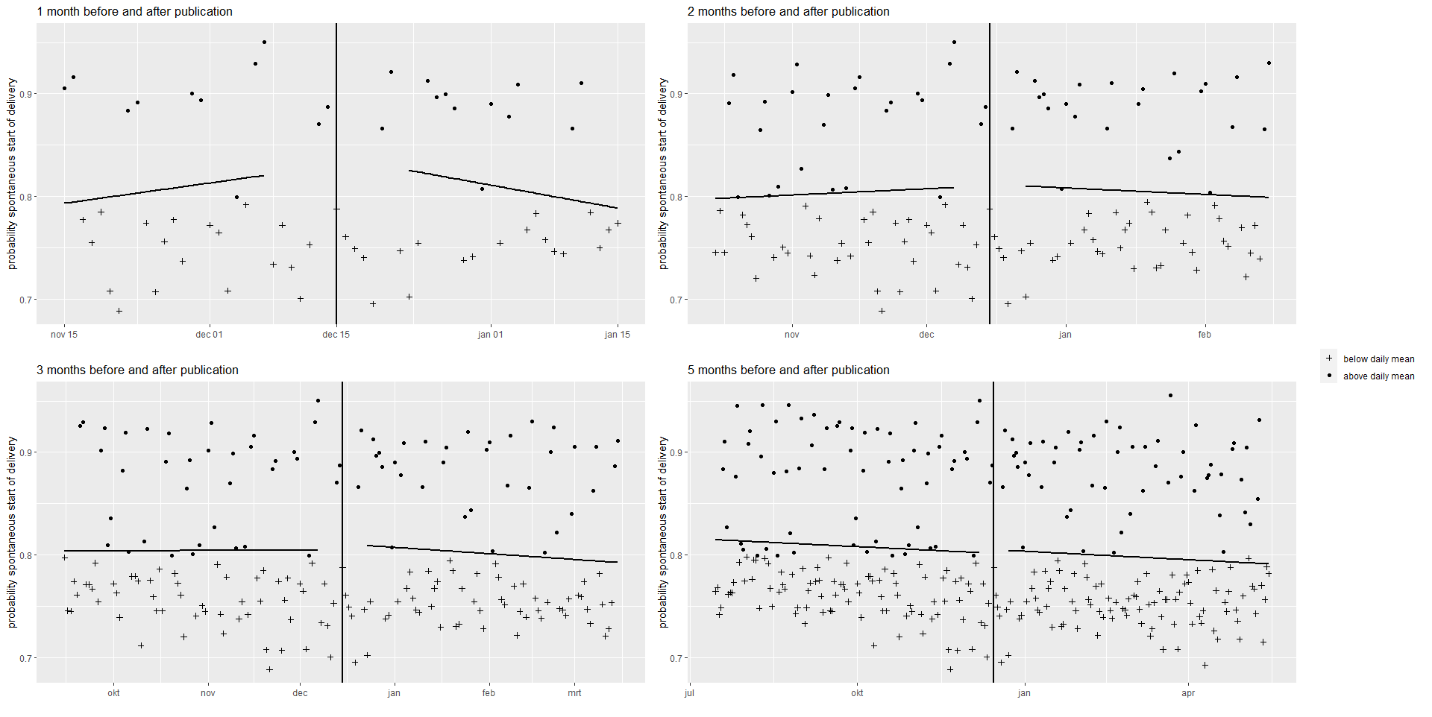
****Supplementary figure 8.*** Probability of a spontaneous start of a delivery plotted against the publication date of the 2008 EURO-PERISTAT report (December 15^th^, 2008) to demonstrate discontinuity at the cut-off value.

***Supplementary figure 9.*** Probability of a spontaneous start of delivery plotted against the publication date of
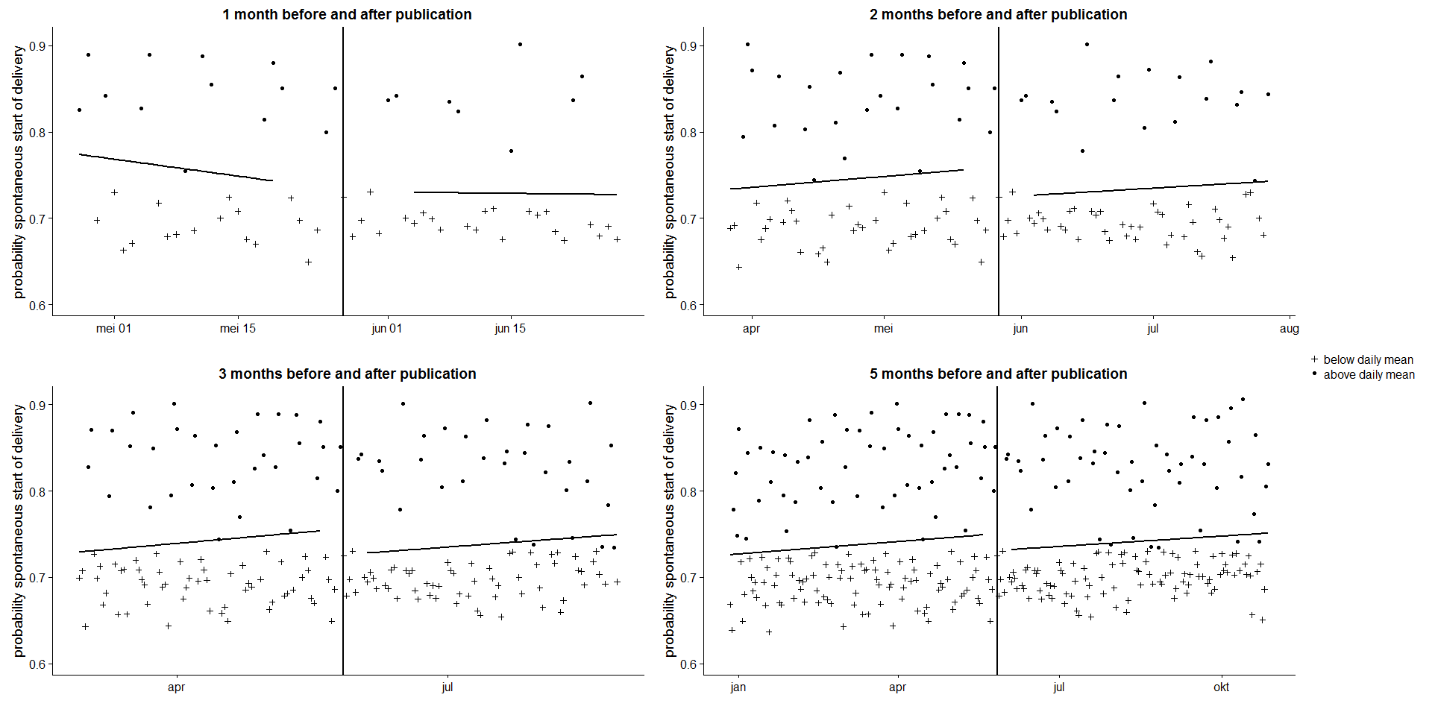
the 2013 EURO-PERISTAT report (May 27^th^, 2013) to demonstrate discontinuity at the cut-off value.

***Supplementary figure 10.*** Probability of an induction of labour plotted against the publication date of the 2003 EURO-PERISTAT report (November 27^th^, 2003) to demonstrate discontinuity at the cut-off value.


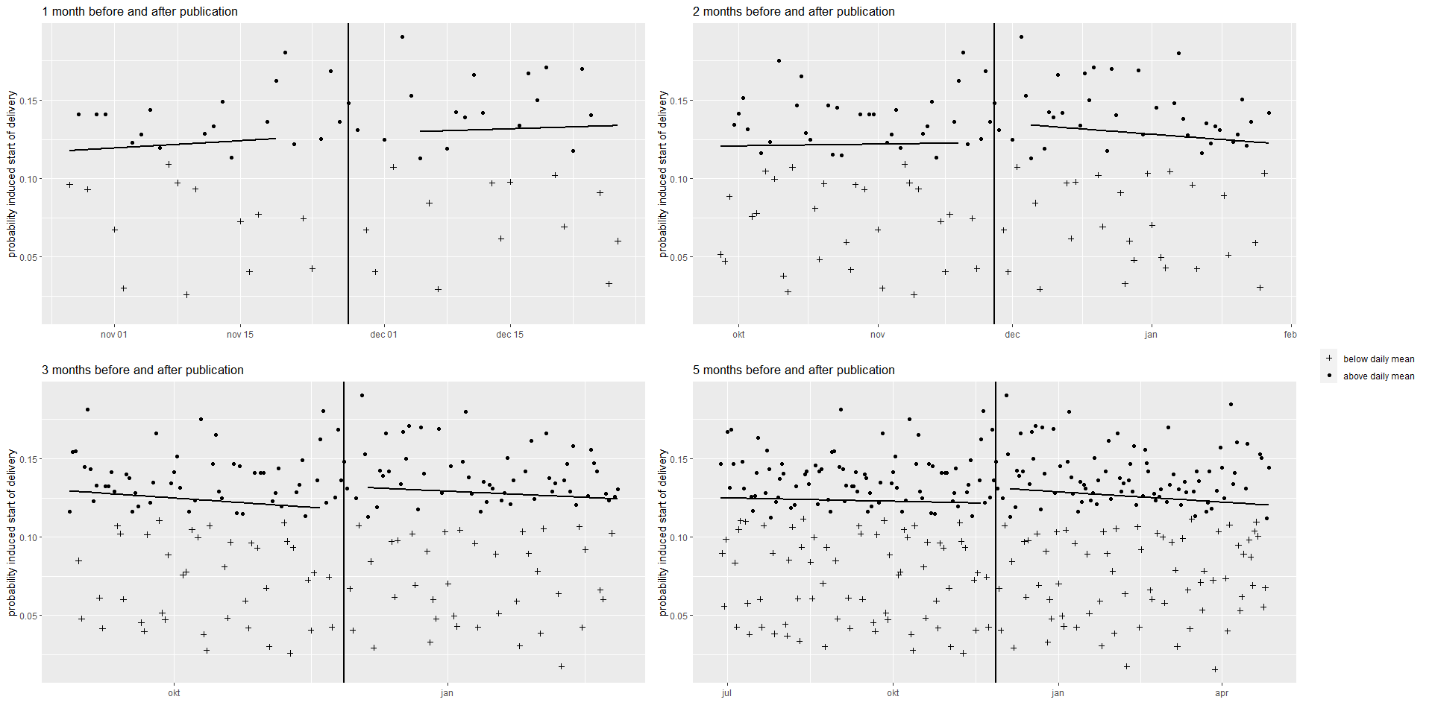


***
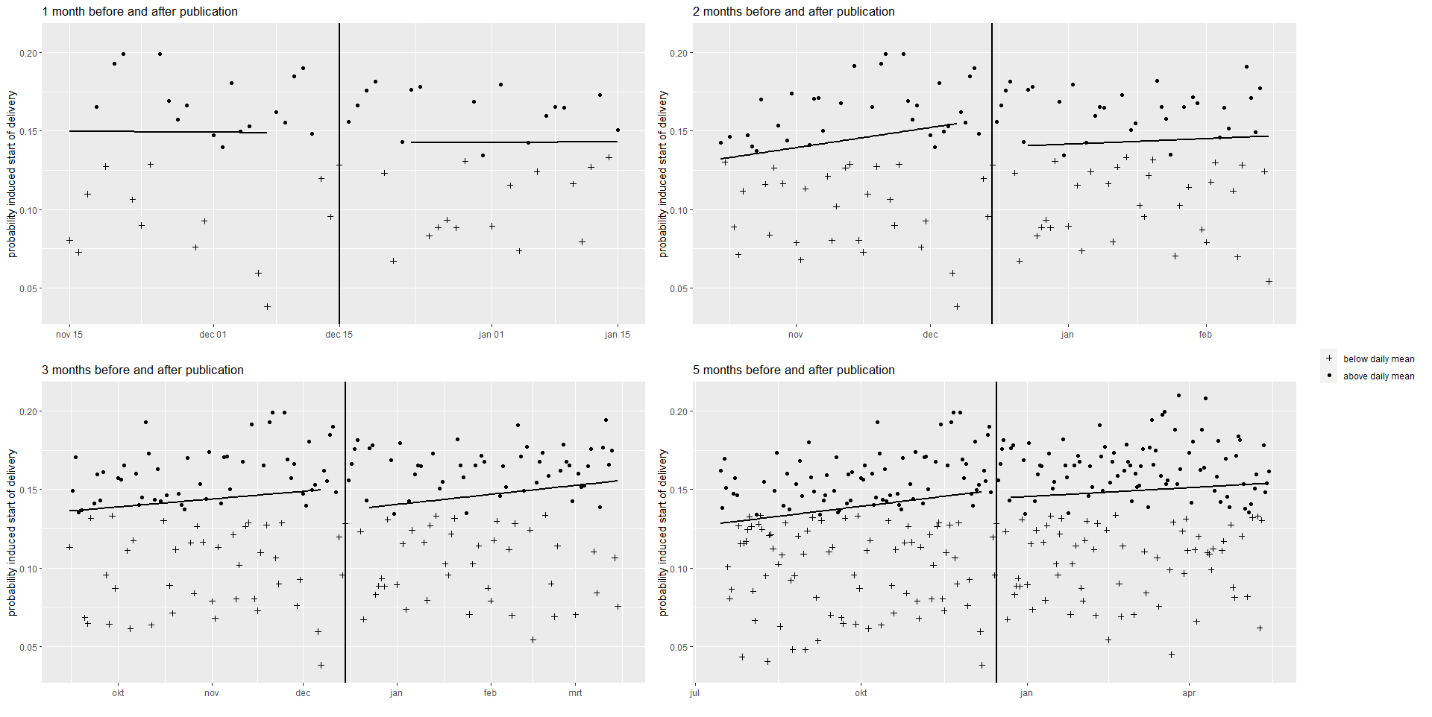
Supplementary figure 11.*** Probability of an induction of labour plotted against the publication date of the 2008 EURO-PERISTAT report (December 15^th^, 2008) to demonstrate discontinuity at the cut-off value.

***Supplementary*** *
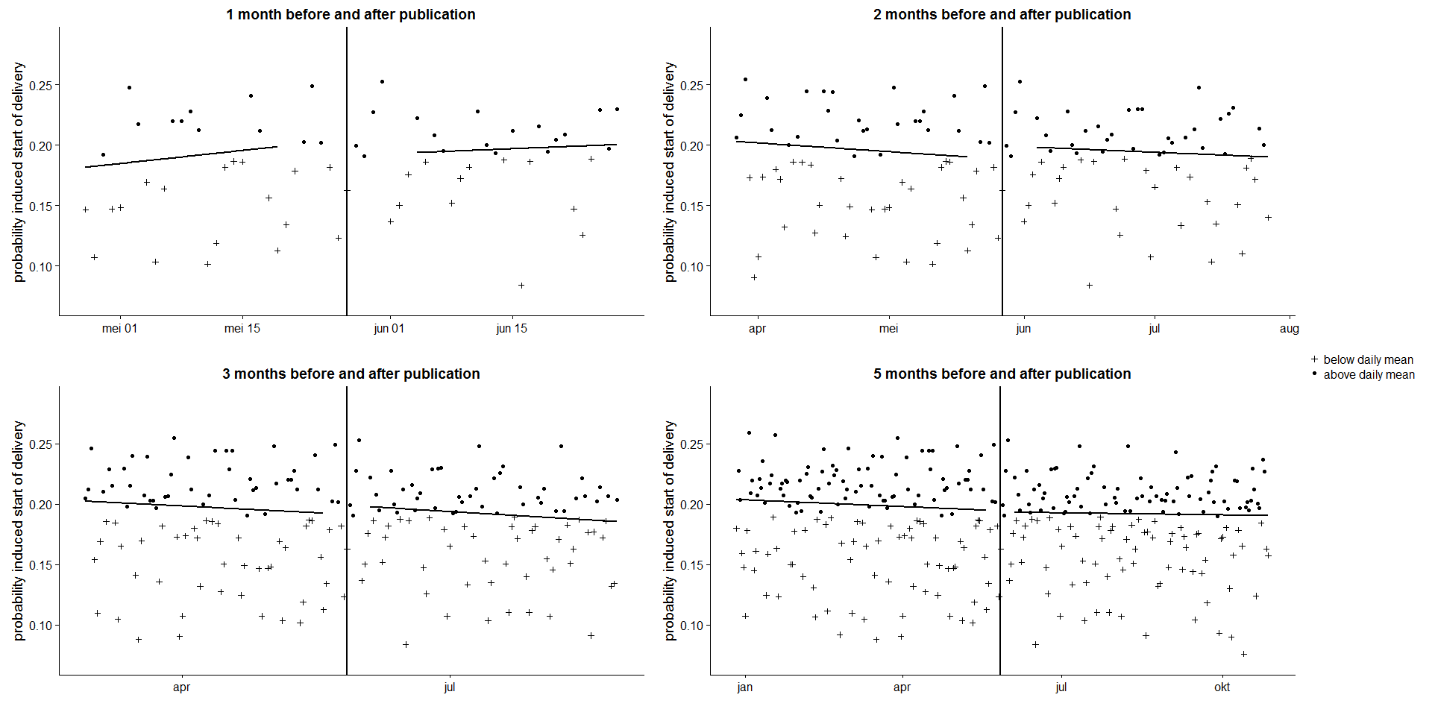
****figure 12.*** Probability of an induction of labour plotted against the publication date of the 2013 EURO-PERISTAT report (May 27^th^, 2013) to demonstrate discontinuity at the cut-off value.

***Supplementary figure 13.*** Probability of a primary caesarean section plotted against the publication date of the 2003 EURO-PERISTAT report (November 27^th^, 2003) to demonstrate discontinuity at the cut-off value.


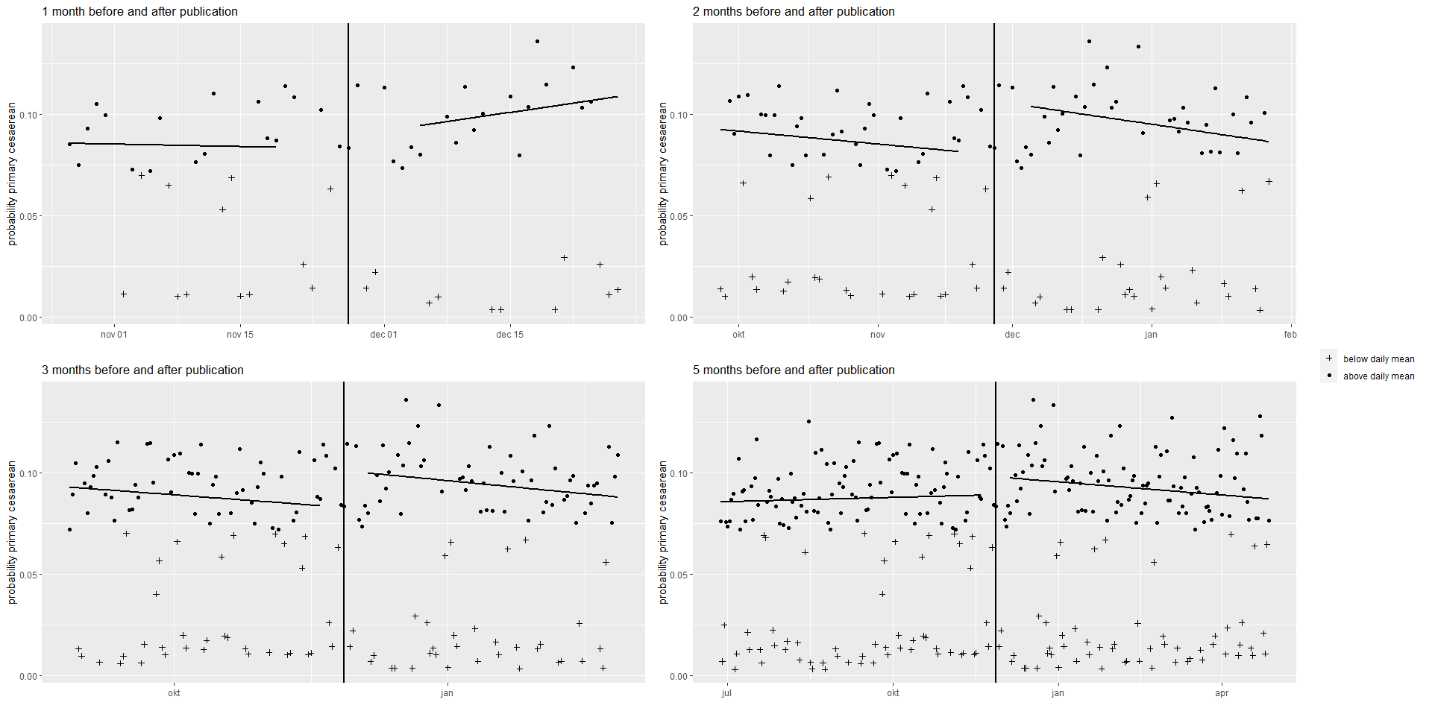


***Supplementary figure 14.*** Probability of a primary caesarean section plotted against the publication date of the 2008 EURO-PERISTAT report (December 15^th^, 2008) to demonstrate discontinuity at the cut-off value.

**
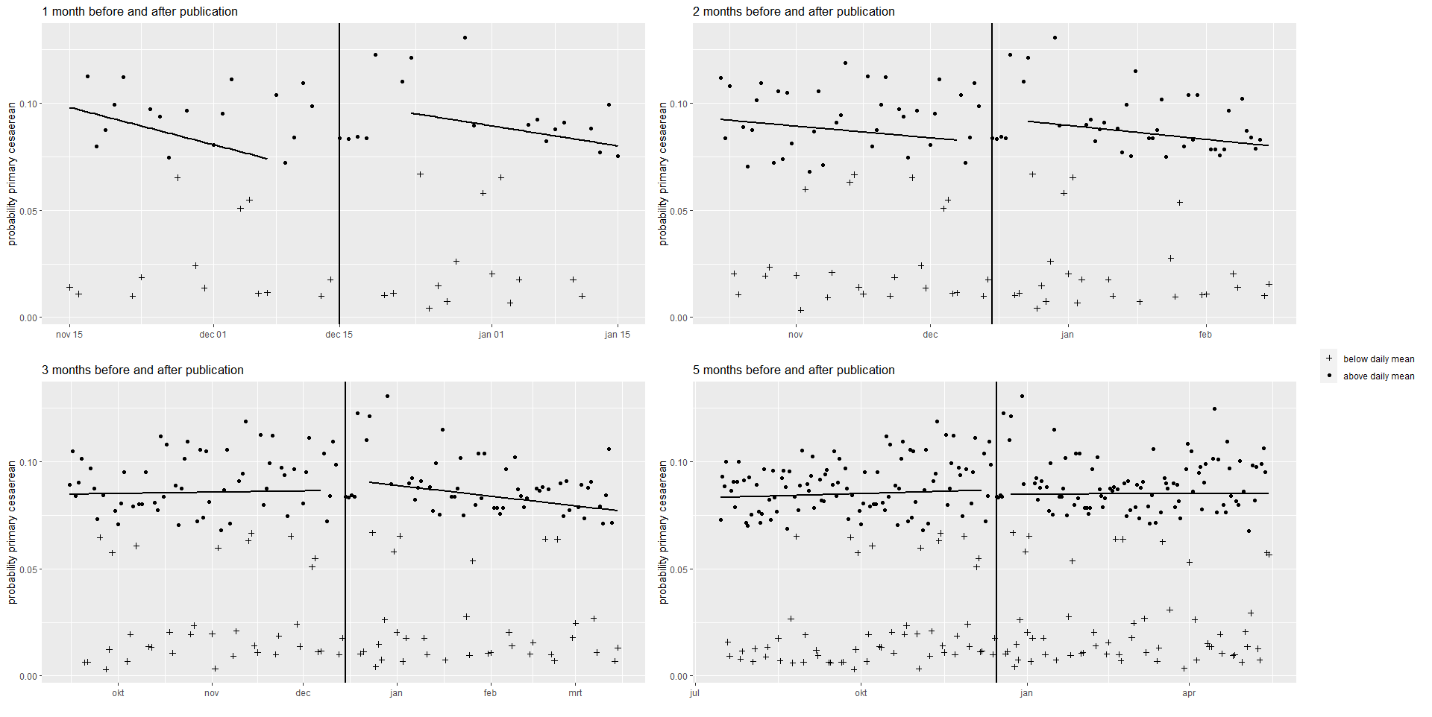
**

***Supplementary figure 15.*** Probability of a primary caesarean section plotted against the publication date of the 2013 EURO-PERISTAT report (May 27^th^, 2013) to demonstrate discontinuity at the cut-off value.

***
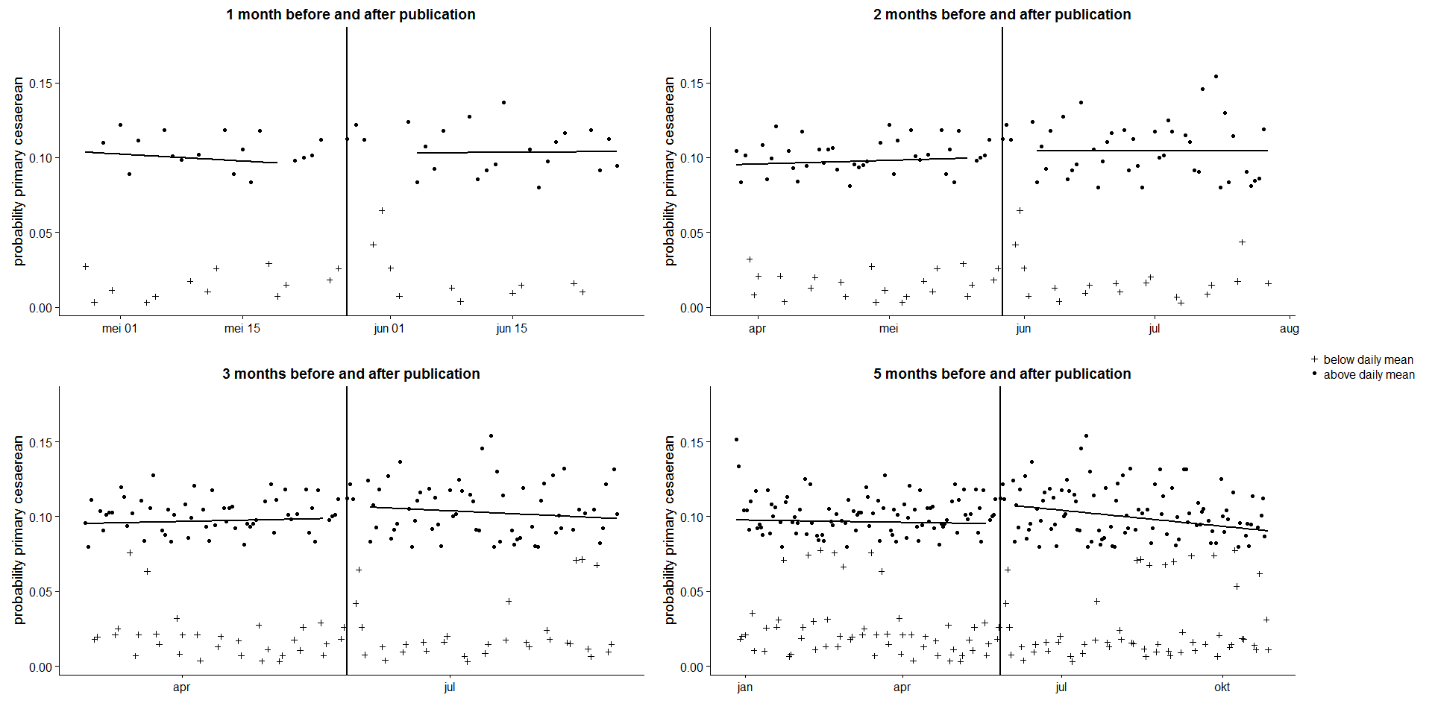
***

***Supplementary figure 16.*** Probability of a spontaneous end of a delivery plotted against the publication date of the 2003 EURO-PERISTAT report (November 27^th^, 2003) to demonstrate discontinuity at the cut-off value.


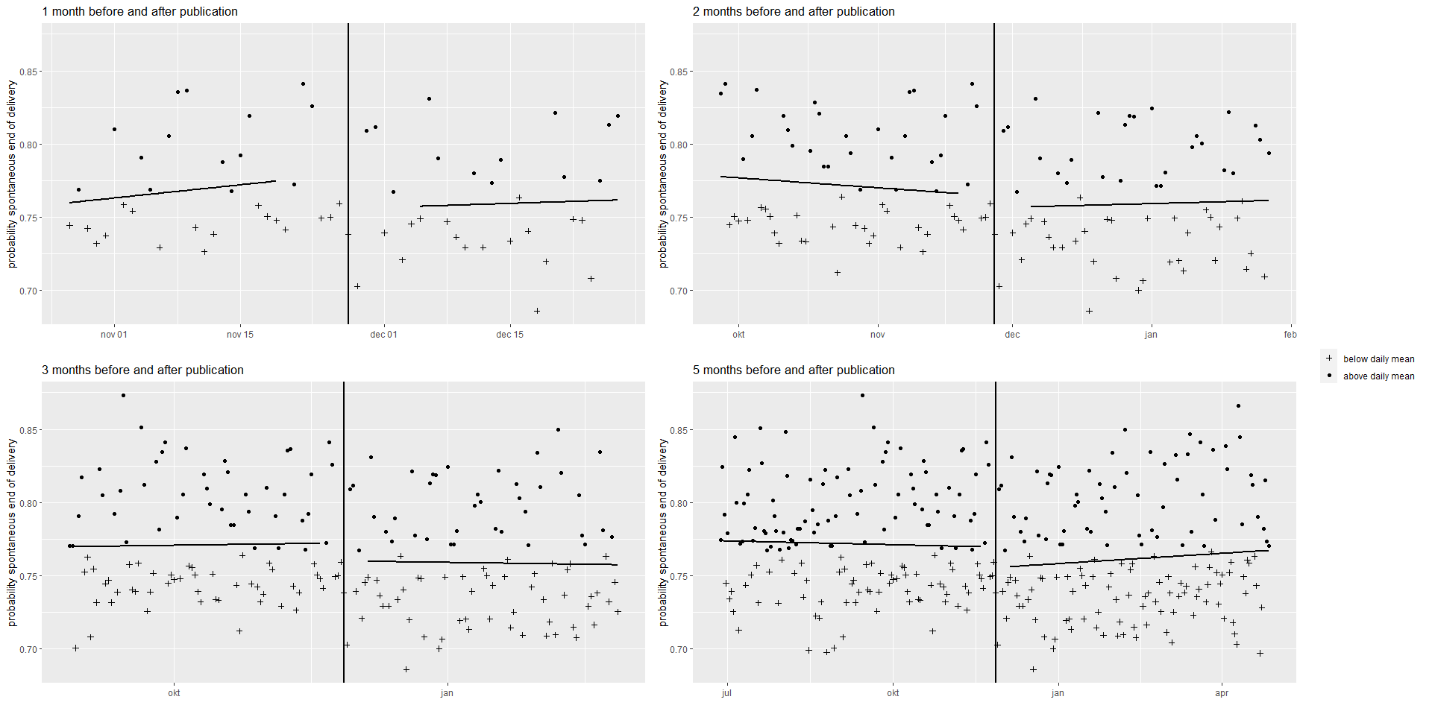


***Supplementary figure 17.*** Probability of a spontaneous end of a delivery plotted against the publication date of the 2008 EURO-PERISTAT report (December 15^th^, 2008) to demonstrate discontinuity at the cut-off value.


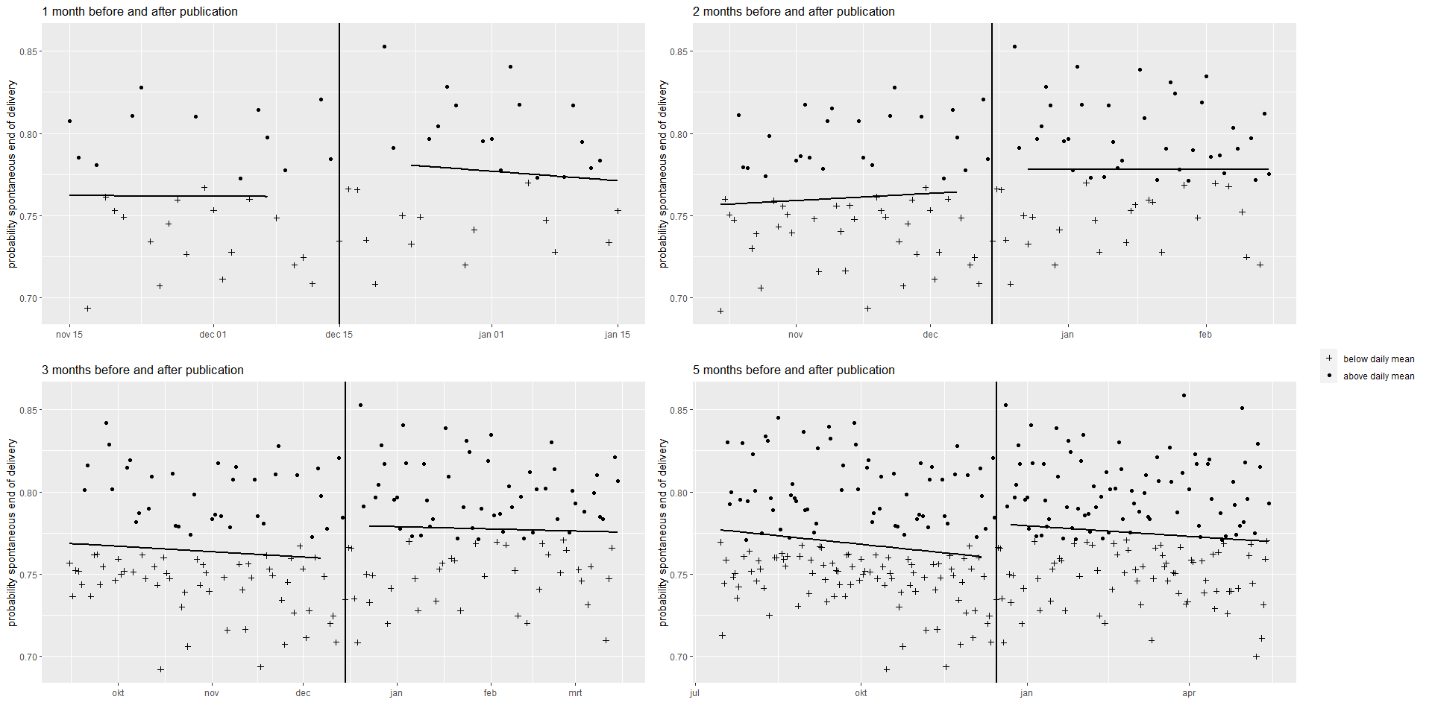


***Supplementary figure 18.*** Probability of a spontaneous end of a delivery plotted against the publication date of the 2013 EURO-PERISTAT report (May 27^th^, 2013) to demonstrate discontinuity at the cut-off value.

***
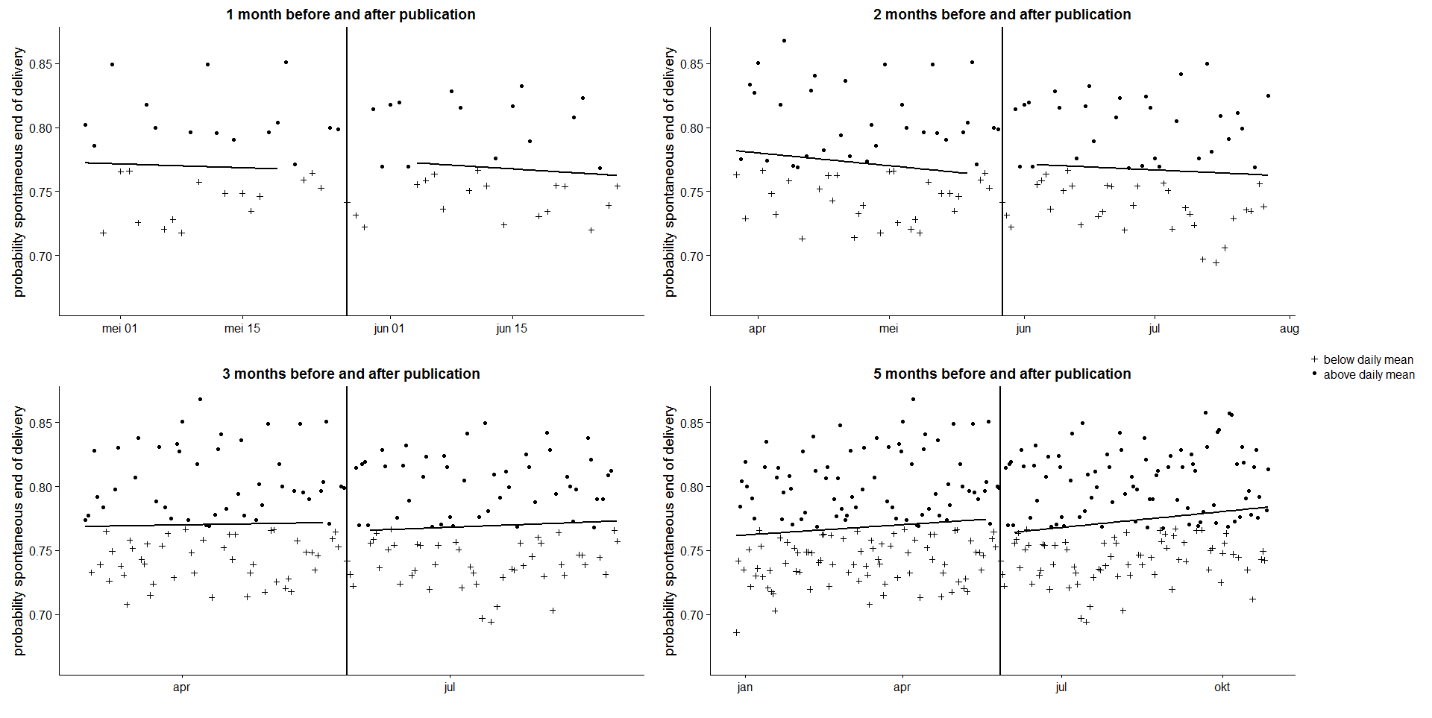
***

***Supplementary figure 19.*** Probability of an assisted vaginal delivery plotted against the publication date of the 2003 EURO-PERISTAT report (November 27^th^, 2003) to demonstrate discontinuity at the cut-off value.


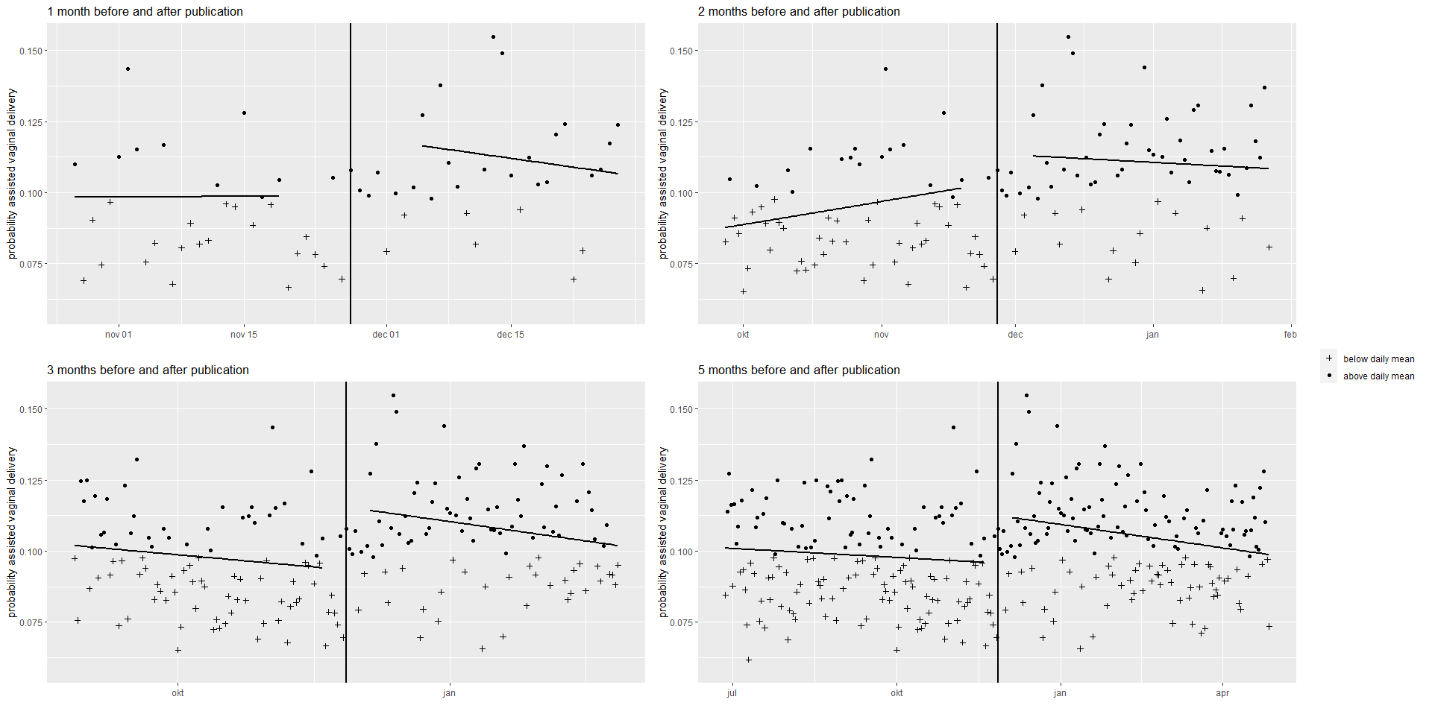


***Supplementary figure 20.*** Probability of an assisted vaginal delivery plotted against the publication date of the 2008 EURO-PERISTAT report (December 15^th^, 2008) to demonstrate discontinuity at the cut-off value.


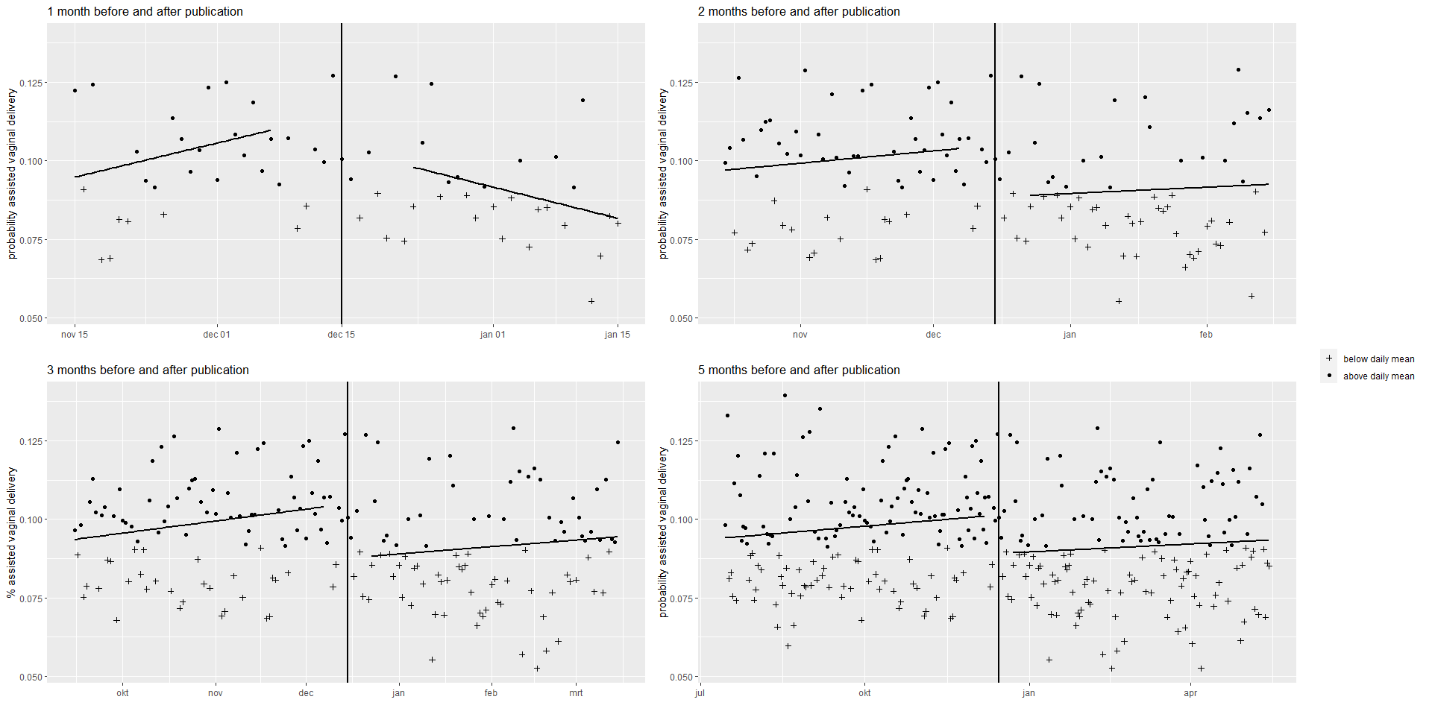


***Supplementary figure 21.*** Probability of an assisted vaginal delivery plotted against the publication date of the 2013 EURO-PERISTAT III report (May 27^th^, 2013) to demonstrate discontinuity at the cut-off **
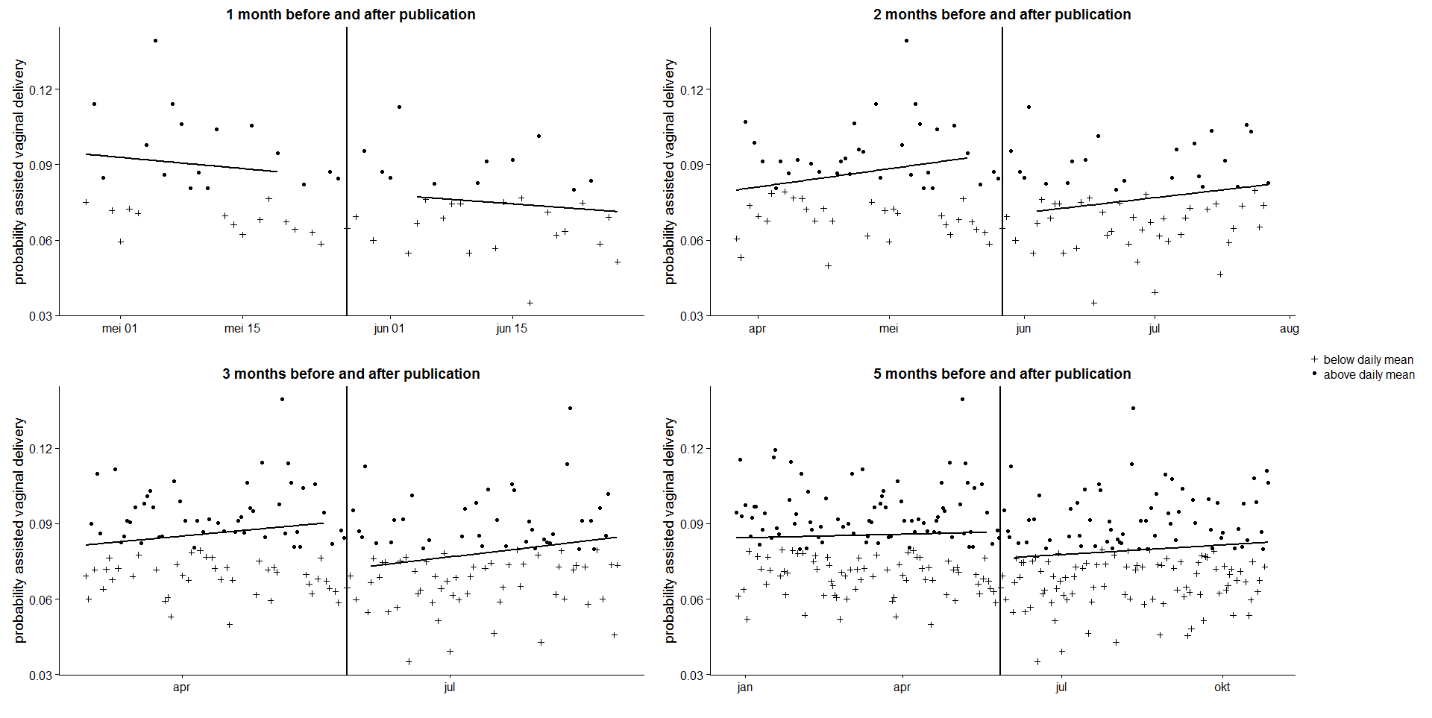
**value.

***Supplementary figure 22.*** Probability of a secondary caesarean section plotted against the publication date of the 2003 EURO-PERISTAT report (November 27^th^, 2003) to demonstrate discontinuity at the cut-off value.

**
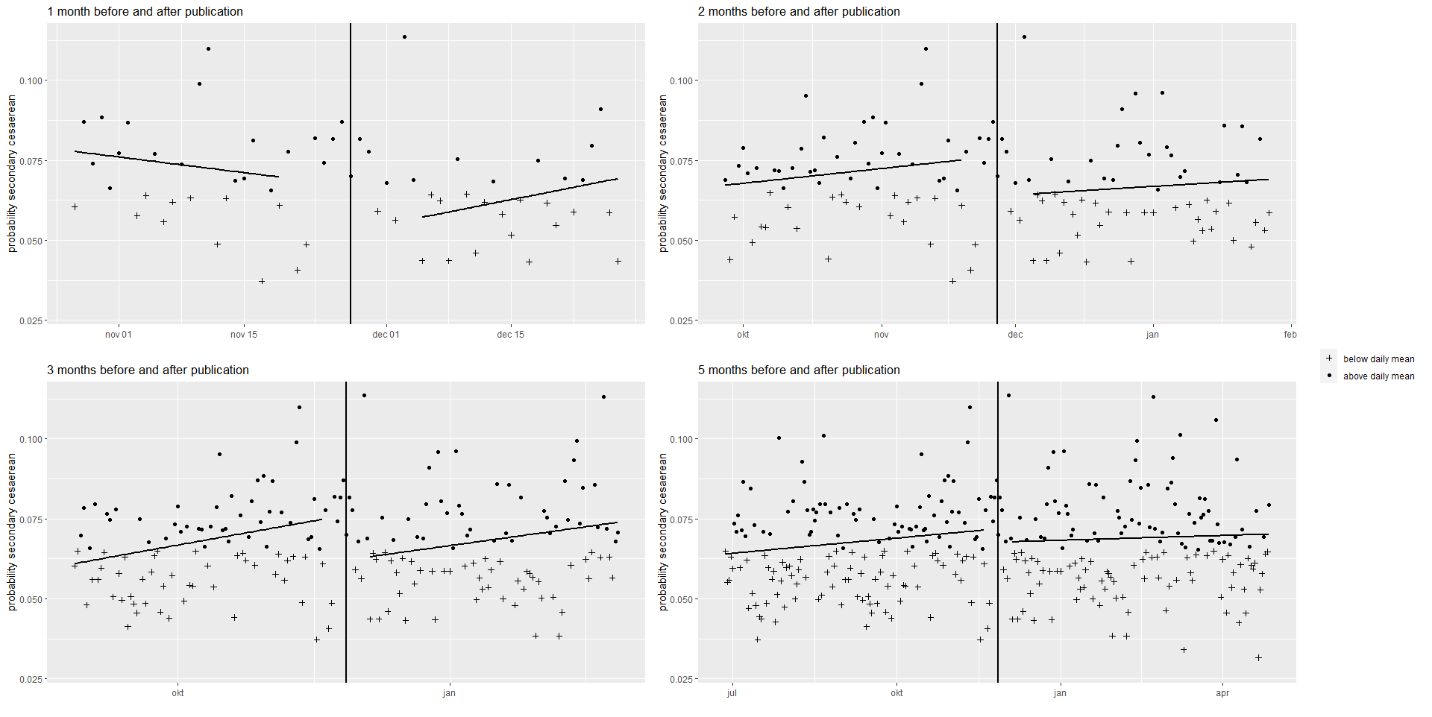
*Supplementary figure 23.*** Probability of a secondary caesarean section plotted against the publication date of the 2008 EURO-PERISTAT report (December 15^th^, 2008) to demonstrate discontinuity at the cut-off value.

**
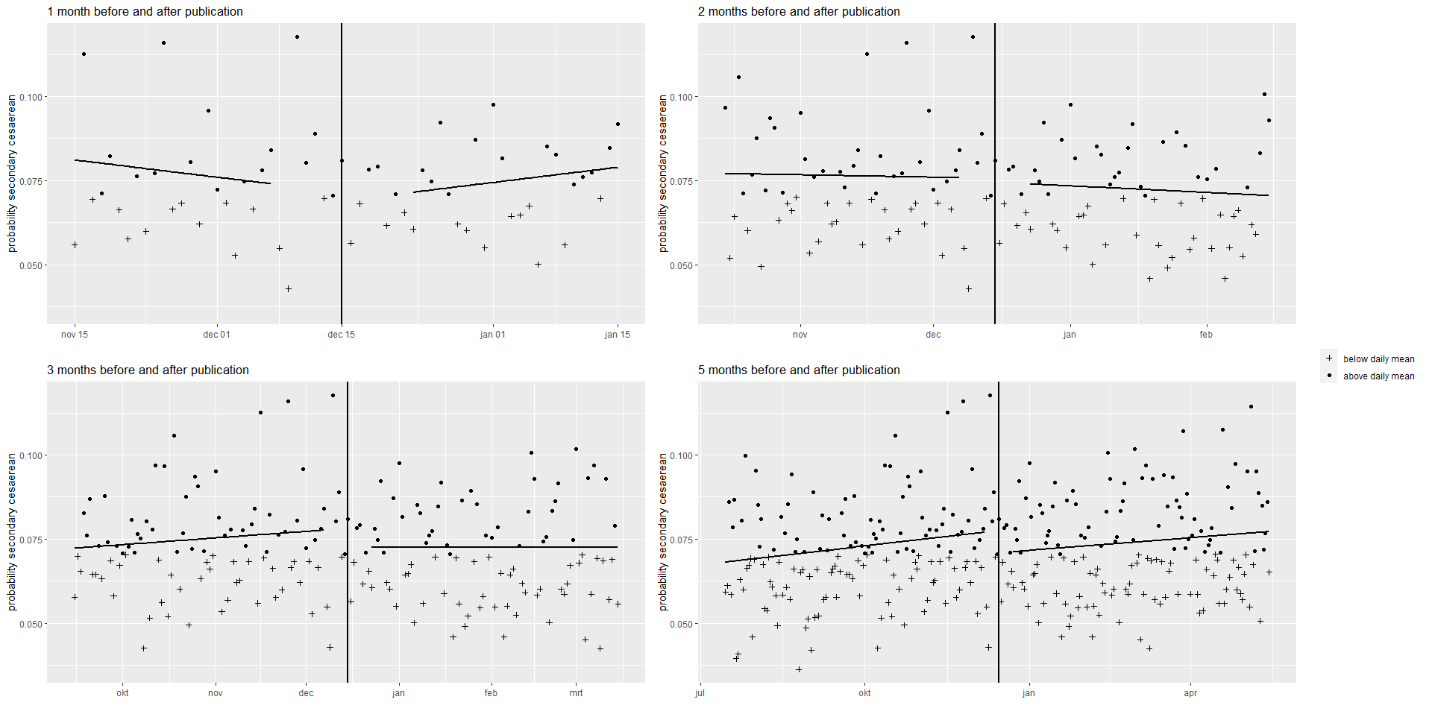
**

***Supplementary figure 24.*** Probability of a secondary caesarean section plotted against the publication date of the 2013 EURO-PERISTAT report (May 27^th^, 2013) to demonstrate discontinuity at the cut-off value.


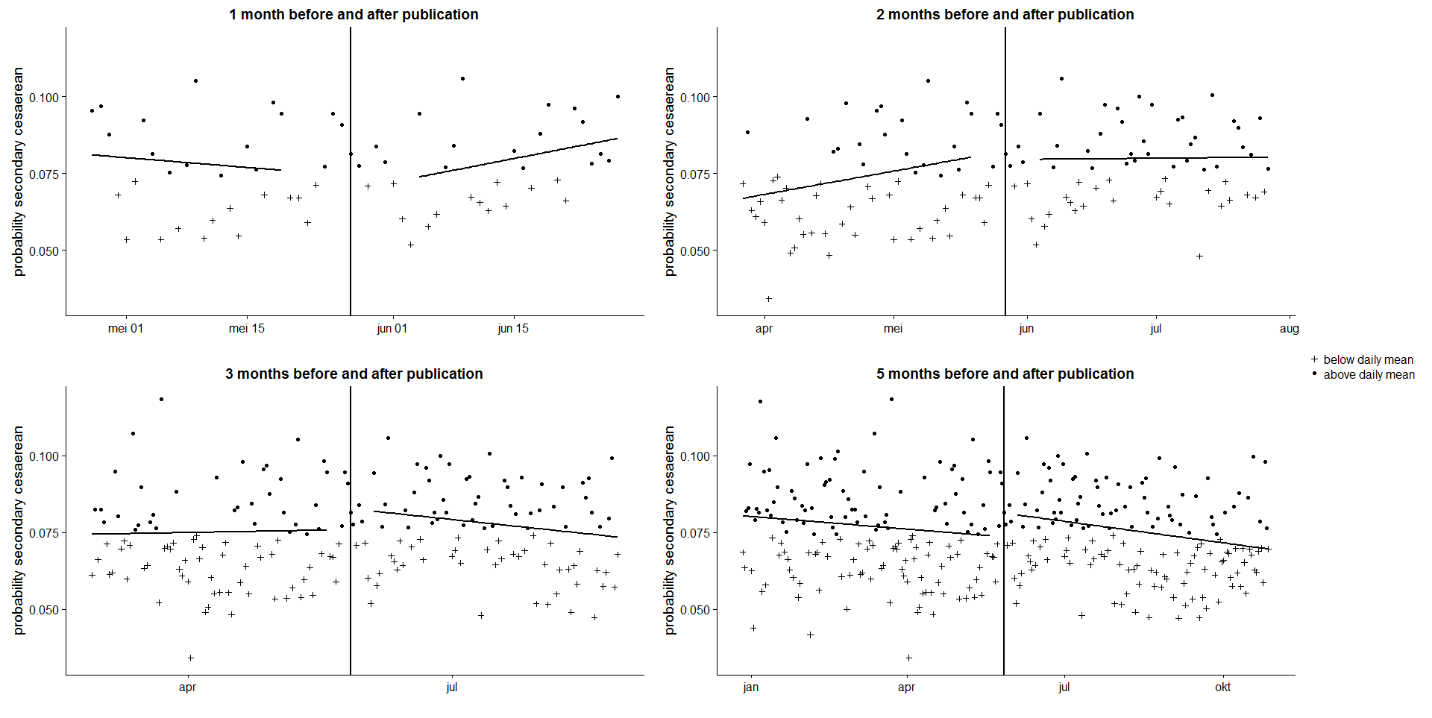


**Supplementary figures 25 and 26.** Incidences of obstetric management at the start and end of a delivery**.**

***Supplementary figure 25.*** Incidences of different obstetric management options at the start of a delivery one year before and one year after publication of the different EURO-PERISTAT reports.


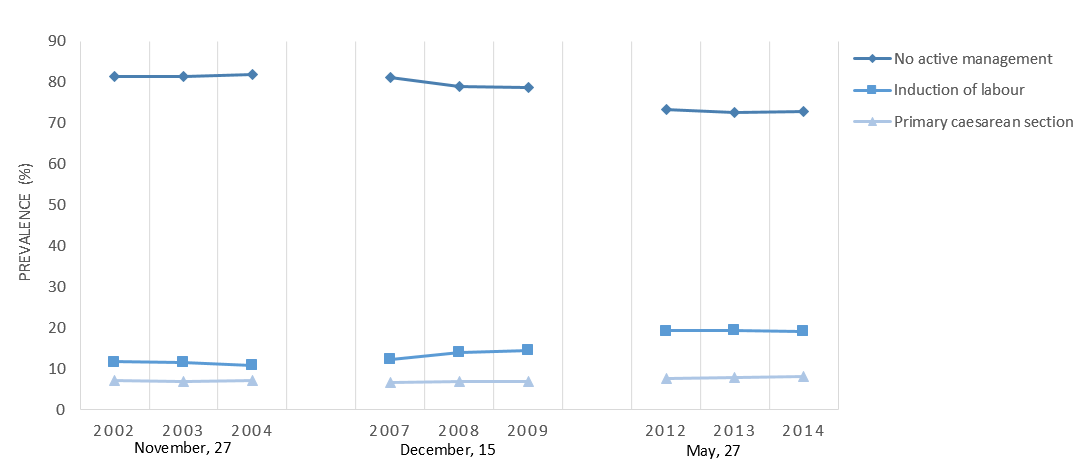


***Supplementary***
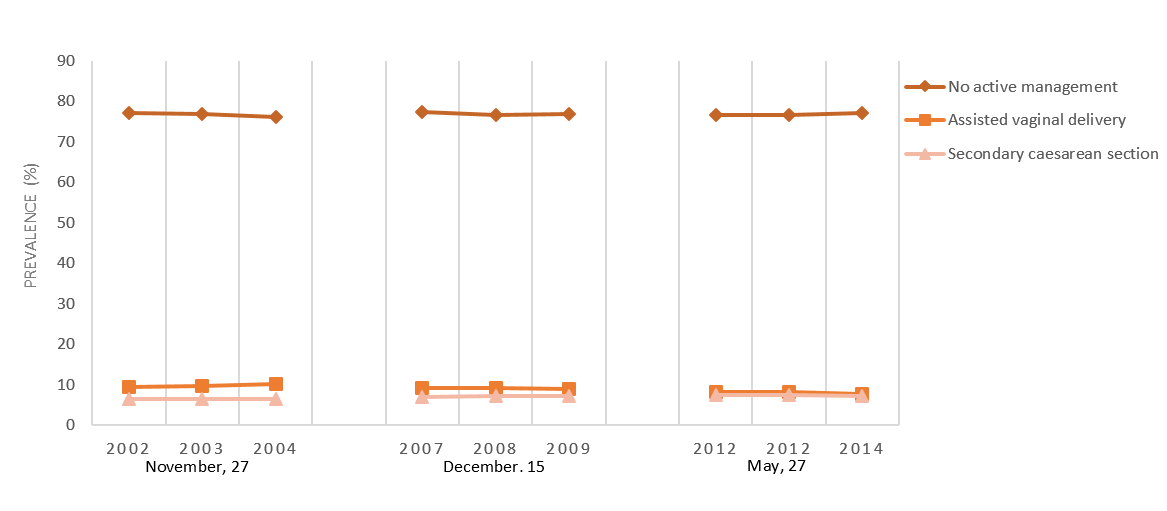
***figure 26.*** Incidences of different obstetric management options at the end of a delivery one year before and one year after publication of the different EURO-PERISTAT reports.

**Supplementary table 1.** Impact of the EURO-PERISTAT 2003 (November 27^th^), 2008 (December 15^th^), and 2013 (May 27^th^) reports on obstetric management at the start and end of a delivery across different time windows before and after publication, without omitting two weeks after the publication dates.

|  | **Time window** | | | |
| --- | --- | --- | --- | --- |
|  | **1-month** | **2-month** | **3-month** | **5-month** |
|  | RR (95% CI)* | RR (95% CI)* | RR (95% CI)* | RR (95% CI)* |
| **Cohort 1:EURO-PERISTAT 2003 analysis **** | n = 124,066 | n =230,243 | n = 338,934 | n = 547,991 |
| *Start of delivery* | | | | |
| Spontaneous | 0.98 (0.95; 1.00) | 0.99 (0.98; 1.01) | 1.00 (0.98; 1.01) | 1.00 (0.99; 1.01) |
| Induction of labour | 1.07 (0.93; 1.22) | 1.03 (0.93; 1.14) | 1.05 (0.96; 1.14) | 1.03 (0.96; 1.11) |
| Planned caesarean section | 1.18 (0.99; 1.40) | 1.01 (0.88; 1.15) | 0.97 (0.86; 1.09) | 0.97 (0.89; 1.07) |
| *End of delivery* | | | | |
| Spontaneous vaginal delivery | 0.98 (0.95; 1.00) | 0.97 (0.89; 1.07) | 0.96 (0.89; 1.04) | 0.93 (0.87; 0.99) |
| Assisted vaginal delivery | 1.17 (1.01; 1.36) | 1.11 (0.97; 1.26) | 1.21 (1.08; 1.35) | 1.22 (1.11; 1.33) |
| Emergency caesarean section | 0.89 (0.74; 1.07) | 0.93 (0.80; 1.08) | 0.90 (0.79; 1.02) | 0.98 (0.88; 1.08) |
| **Cohort 2: EURO-PERISTAT 2008 analysis **** | n =103,312 | n = 210,682 | n = 313,766 | n = 529,258 |
| *Start of delivery* | | | | |
| Spontaneous | 1.01 (0.98; 1.04) | 1.00 (0.98; 1.02) | 1.00 (0.98; 1.01) | 1.00 (0.99; 1.01) |
| Induction of labour | 0.95 (0.83; 1.09) | 0.96 (0.87; 1.06) | 1.04 (0.91; 1.07) | 0.99 (0.93; 1.06) |
| Planned caesarean section | 1.05 (0.86; 1.29) | 1.07 (0.92; 1.23) | 1.05 (0.93; 1.18) | 1.02 (0.93; 1.12) |
| *End of delivery* | | | | |
| Spontaneous vaginal delivery | 1.02 (0.99; 1.05) | 1.01 (0.99; 1.03) | 1.01 (1.00; 1.03) | 1.02 (1.00; 1.03) |
| Assisted vaginal delivery | 0.90 (0.76; 1.07) | 0.90 (0.79; 1.01) | 0.88 (0.80; 0.98) | 0.89 (0.82; 0.97) |
| Emergency caesarean section | 0.91 (0.75; 1.11) | 0.97 (0.85; 1.12) | 0.96 (0.85; 1.08) | 0.94 (0.86; 1.04) |
| **Cohort 3: EURO-PERISTAT 2013 analysis **** | n = 107,224 | n = 218,290 | n = 324,380 | n = 541,390 |
| *Start of delivery* | | | | |
| Spontaneous | 0.99 (0.96; 1.03) | 0.97 (0.95; 0.99) | 0.97 (0.95; 0.99) | 0.98 (0.96; 0.99) |
| Induction of labour | 1.00 (0.89; 1.13) | 1.06 (0.98; 1.15) | 1.04 (0.98; 1.12) | 1.03 (0.97; 1.09) |
| Planned caesarean section | 1.06 (0.87; 1.28) | 1.15 (1.00; 1.32) | 1.17 (1.05; 1.32) | 1.15 (1.06; 1.26) |
| *End of delivery* | | | | |
| Spontaneous vaginal delivery | 0.99 (0.96; 1.02) | 1.00 (0.98; 1.02) | 0.99 (0.97; 1.01) | 0.99 (0.97; 1.00) |
| Assisted vaginal delivery | 1.07 (0.88; 1.29) | 0.90 (0.79; 1.03) | 0.89 (0.79; 0.99) | 0.91 (0.83; 0.99) |
| Emergency caesarean section | 0.94 (0.77; 1.15) | 0.97 (0.85; 1.12) | 1.08 (0.96; 1.21) | 1.10 (1.00; 1.20) |

*All analyses were adjusted for maternal age, parity, ethnicity and socioeconomic status. ** The reference group is the obstetric management option in the same time window before publication (e.g. RR for induction of labour in the 1-month time window shows the odds for a labour induction 1-month after the publication compared to 1-month before the publication).
